# EPR spectroscopy reveals glycerol-dependent activation of cysteamine dioxygenase (ADO) enables bidentate substrate coordination

**DOI:** 10.1016/j.jbc.2026.111438

**Published:** 2026-04-14

**Authors:** Joshua R. Helms, Miriam Probst, Jared Paris, Patrycja Szamweber, Zhitao Zhao, Si Wu, Brad S. Pierce

**Affiliations:** 1 Department of Chemistry & Biochemistry, University of Alabama, Tuscaloosa, Alabama, USA; 2 Department of Chemistry, Carnegie Mellon University, Pittsburgh, Pennsylvania, USA

**Keywords:** computational, DFT, EPR spectroscopy, molecular crowding, mononuclear non-heme iron oxygenase, N-terminal cysteinyl oxygenase, thiol dioxygenase

## Abstract

In mammals, cysteine dioxygenase (CDO) and cysteamine dioxygenase (ADO) are the only known thiol dioxygenases (TDOs). These enzymes employ a mononuclear non-heme iron active site to catalyze the O_2_-dependent oxidation of L-cysteine (CYS) and cysteamine (CA), producing cysteine sulfinic acid (CSA) and hypotaurine (HT), respectively. Notably, ADO also exhibits N-terminal cysteinyl dioxygenase (NCO) activity, oxidizing protein N-terminal cysteine (N_t_-CYS) residues to initiate N-end rule degradation of G-protein regulators. Thus, ADO functions both as a small-molecule thiol dioxygenase and as an O_2_-dependent regulator of protein stability. Although TDO substrates typically bind the ferrous iron active site in a bidentate manner, the nature of substrate coordination at the ADO Fe-site is debated. Here, we show that molecular crowding by glycerol shifts the CA binding equilibrium toward bidentate coordination at the ADO Fe site, matching the coordination observed for the N_t_-CYS RGS5 polypeptide. Continuous-wave and pulsed EPR spectroscopy (ESEEM) show that glycerol occupies the outer coordination sphere of the Fe-site and stabilizes bidentate CA binding *via* a thiolate and neutral amine. Concomitant with this shift toward bidentate substrate coordination is a ∼7-fold increase in catalytic efficiency, suggesting that this coordination environment represents the catalytically relevant O_2_-activating enzyme–substrate complex. Supporting spectroscopic measurements (CD and Mössbauer) and computational DFT models are consistent with enzymatic activation being triggered by a switch in substrate-binding denticity rather than by changes in protein secondary structure. Together, these results identify glycerol as a conformational activator of ADO and resolve the ambiguous nature of substrate-binding denticity at the enzymatic Fe-site.

Thiol dioxygenases (TDOs) are mononuclear non-heme iron enzymes that catalyze the O_2_-dependent oxidation of thiol-containing substrates to yield sulfinic acids. In mammals, two TDOs have been identified: (1) cysteine dioxygenase (CDO, E.C. 1.13.11.20) and (2) cysteamine dioxygenase (ADO, E.C. 1.13.11.19). CDO and ADO convert free L-cysteine (CYS) and cysteamine (CA) into cysteine sulfinic acid (CSA) and hypotaurine (HT), respectively (1, 2). Both enzymatic products are intermediates in the major biosynthetic route to taurine (TAU) (3), an abundant sulfonic acid derivative involved in osmoregulation, cardiac and skeletal muscle stabilization, and neurotransmission (3, 4, 5). Although CDO and ADO act on structurally similar substrates, they display strict substrate specificity with negligible cross-reactivity (1). These enzymes are not uniformly expressed throughout the body; therefore, the primary route of TAU biosynthesis depends on the relative concentrations of ADO and CDO present in the cell (1, 6). Consequently, dysregulation of either enzyme has been linked to oxidative stress and pathologies including cancer, neurodegenerative disorders, and inflammatory diseases, thereby underscoring the physiological importance of sulfur oxidation (6, 7, 8, 9, 10, 11, 12, 13).

An emerging subcategory of TDOs (referred to here as NCOs) exhibits N-terminal cysteinyl dioxygenase activity (14). NCOs function as eukaryotic oxygen sensors by catalyzing the oxidation of protein N-terminal CYS residues (N_t_-CYS) to yield a protein sulfinate on target proteins. This post-translational modification initiates the Cys/Arg-N-degron pathway, which ultimately leads to ubiquitination and proteasomal degradation (15). Plant cysteine oxidases (PCOs), which target the N_t_-CYS residue on the ethylene response factor VII (ERF-VII) transcription factor under aerobic conditions, is a well-characterized example (16, 17, 18). Similarly, it has been demonstrated that mammalian ADO targets N_t_-CYS residues of regulators of G-protein signaling (19). Thus, ADO appears to serve as a dual-function oxygenase, capable of catalyzing HT production to feed into the TAU biosynthetic pathway, and as an NCO involved in the O_2_-dependent regulation of protein stability.

Members of the small-molecule thiol dioxygenases (smTDOs) include CDO (2, 8, 20), 3-mercaptopropionate dioxygenase (MDO) (21, 22, 23, 24), and mercaptosuccinate dioxygenase (MSDO) (25), all of which share a conserved CDO_I (PF05995) fold and a 3-His facial triad that coordinates the mononuclear ferrous iron site (26). As shown in Figure 1*A*, smTDOs contain a highly conserved Ser-His-Tyr sequence, referred to as the SHY motif, that forms a hydrogen-bonding network enhancing both catalytic turnover and substrate binding (27, 28, 29). Notably, a recent study demonstrated that Tyr159 in the MDO SHY motif donates a hydrogen bond to the O_2_-binding site, conferring nearly a 20-fold increase in *k*_cat_ (30). In mammalian CDO, this motif undergoes a unique post-translational modification in which Tyr157 forms a covalent cross-link with Cys93 (31), substantially enhancing catalytic efficiency (27, 28, 32, 33). Crystallographic and spectroscopic studies have shown that smTDO substrates bind bidentate to the Fe site through their thiolate and amine (or carboxylate) functional groups. This coordination confers high regio-, chemo-, and stereoselectivity (27, 34).

**Figure 1:**
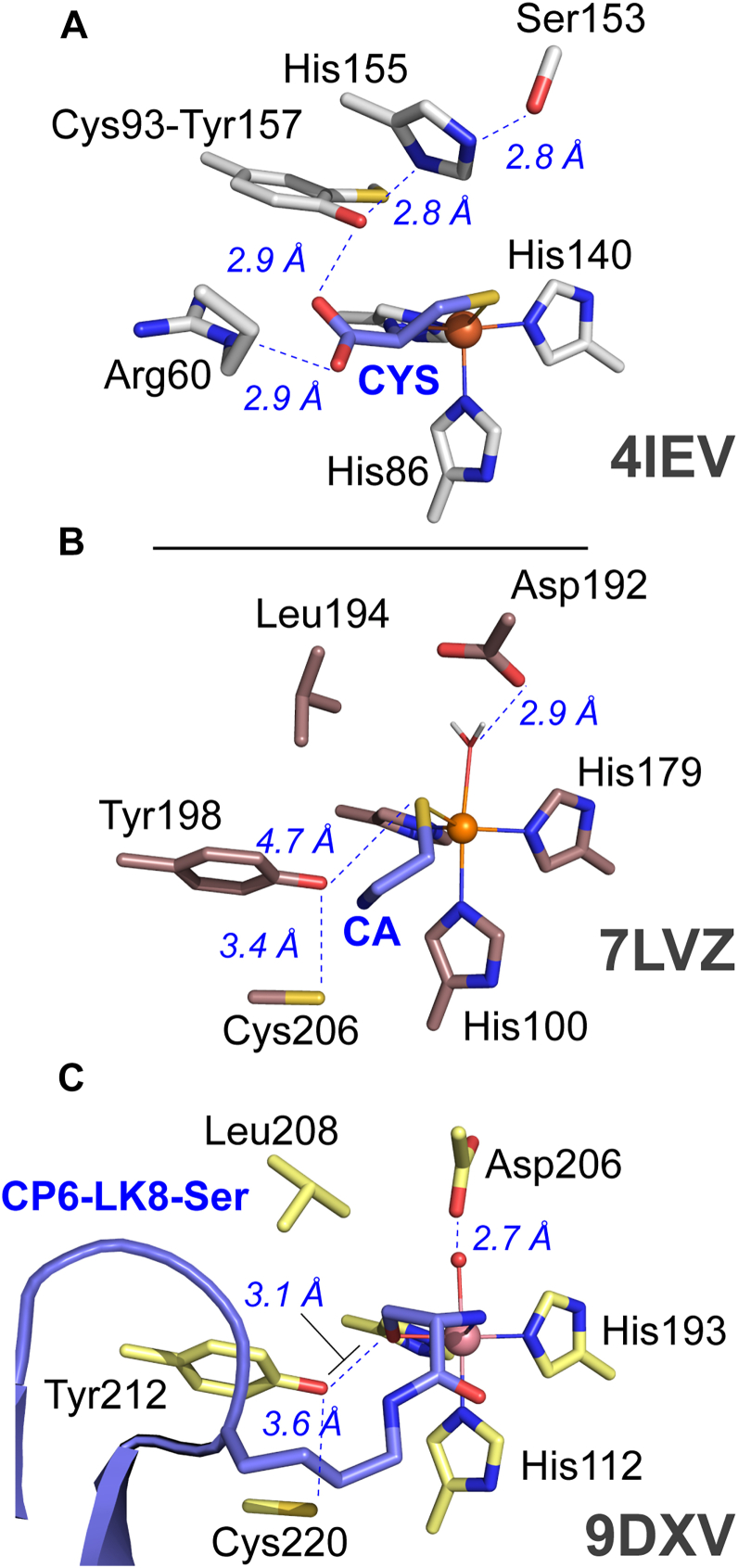
**Comparison of thiol dioxygenase substrate-bound structures.** Aligned active site structures and selected distances for mammalian CDO (*A*, PDB: 4IEV) and ADO (*B*, PDB: 7LVZ; *C*, PDB: 9DXV). The monodentate CA-bound ADO structure (*panelB*) is based on QM/MM computational modeling of the resting enzyme structure (PDB: 7LVZ). *PanelC* shows the XRD structure for Co(II)-substituted human ADO co-crystallized with a cyclic peptide (CP6-LK8-Ser). In all structures, the substrate is shown in *blue*.

By contrast, NCOs are a unique subset of TDOs [Pfam (PF07847)] and have low sequence similarity to smCDOs (26). Multiple crystal structures have been reported for the resting, substrate-free, ADO (35, 36, 37). Like their smTDO analogues, NCOs have a mononuclear non-heme iron active site coordinated by three protein-derived His residues (Fig. 1*B*). However, the SHY proton relay network is absent in the outer (or second) coordination sphere. Instead, this triad is replaced by an (X-Asp-Leu) sequence, resulting in the placement of an anionic Asp192 residue 2.9 Å from the Fe-site. However, structures for ADO in complex with either substrate (CA or RGS) are unavailable. Significantly, the active site Asp residue is conserved among NCOs and appears to be essential for catalysis (37, 38, 39). However, as indicated by the structures shown in Figure 1, *B* and *C*, there is considerable debate in the literature regarding the manner in which CA and the N_t_-CYS residue of RGS bind to the enzymatic Fe-site (26, 35, 36, 37, 40).

Spectroscopic studies suggest that the denticity of CA-binding at the ADO Fe-site is monodentate, representing a significant point of deviation among characterized smTDOs. In one such study, magnetic circular dichroism (MCD) spectroscopy was used to investigate the binding of CA and CYS to the catalytically inert, oxidized form of ADO [Fe(III)-ADO] (40). Here, Fernández *et al.* observed that CA binds to the oxidized Fe(III)-site through thiolate coordination only. This study was used to benchmark the QM/MM structure shown in Figure 1*B*, revealing monodentate coordination of CA-thiolate *trans* to His179. In this same work, it was also reported that CA and CYS coordinate to the Fe(III)-site in different geometries, offering a possible mechanism by which these structurally similar substrates are differentiated by the enzyme. Shortly after publication of this MCD study, Wang *et al.* used EPR spectroscopy to investigate the binding of nitric oxide (NO), CA, and RGS5, an N_t_-CYS polypeptide (CKGLAALPHSCLER) corresponding to the N-terminal sequence of a regulator of G-protein signaling. While the conclusions presented in this report were mixed, the authors suggested that the signals observed were consistent with thiol-only binding of CA, but RGS5 showed some indication of bidentate coordination similar to CDO (41, 42).

In direct contradiction to the above spectroscopic and computational work, Jiramongkol *et al.* recently reported the crystal structure (Fig. 1*C*) for cobalt(II)-substituted ADO in complex with a cyclic peptide (CP6-LK8-Ser) produced by mRNA display, which reveals bidentate coordination of an N_t_-Ser residue to the ADO Fe-site (37). Although not a viable substrate, CP6-LK8-Ser exhibits tight binding to ADO (21 ± 5 nM) and inhibits ADO catalysis. Based on this structure, the authors argue that the N_t_-CYS thiolate replaces the serine hydroxyl group, coordinating *trans* to His193. This is equivalent to the position reported for the CA thiolate predicted by the QM/MM computational model (Fig. 1*B*). This places the N_t_-amino group of CYS coordinating *trans* to His114 and the putative O_2_-binding site *trans* to His112. Significantly, this would position iron-oxo intermediates produced during catalytic turnover within 2.7 to 2.9 Å of the anionic and catalytically essential Asp206 residue (37).

In a seemingly unrelated observation, it was demonstrated by EPR spectroscopy that inclusion of the common cryoprotectant, glycerol, altered the binding of CYS from monodentate to bidentate at the oxidized Fe(III)-site of ADO (40). Of note, a similar glycerol-dependent shift in binding denticity was not observed for the catalytically relevant CA-substrate. This observation suggested that either conformational rearrangements within ADO or molecular crowding effects introduced by glycerol can influence binding at the enzymatic Fe-site. Since MCD studies often require high concentrations of glycerol to improve the glassing properties of samples, this observation is potentially impactful when interpreting MCD results for the enzyme-substrate complex. Another consideration is that the experiments were performed on the catalytically inert, oxidized enzyme [Fe(III)-ADO]. Consequently, the impact of this glycerol-dependent conformational change on catalytic activity was not examined.

Molecular crowding and solvent viscosity exert a significant yet incompletely defined influence on enzyme catalysis. In the cellular environment, the high concentration of macromolecules limits solvent accessibility and alters the physicochemical properties of the medium, thereby modulating protein conformational equilibria and reaction kinetics. However, the effects of crowding on enzymatic turnover are strongly context dependent, reflecting a complex interplay between excluded volume, microviscosity, and specific crowder–protein interactions. While global trends are well-recognized, mechanistic insight into how increased viscosity and crowding perturb active-site geometry, solvent structure, and dynamic sampling remains limited. For example, glycerol is often observed to alter enzymatic behavior, yet most studies report only phenomenological effects without revealing the molecular interactions that drive changes in reactivity (43, 44, 45, 46, 47, 48, 49). In this work, continuous-wave (CW) and pulsed EPR spectroscopy, complemented by Mössbauer and solvent kinetic viscosity effects (SKVE), were used to investigate solvent-dependent changes in substrate binding. Glycerol was found to associate within the outer coordination sphere of the ADO active site, promoting a shift from monodentate to bidentate CA-coordination at the enzymatic Fe-site and resulting in a ∼7-fold increase in catalytic efficiency. While other micro- and macroviscogens were examined, this effect was only observed for glycerol. Crucially, bidentate coordination of the N_t_-CYS RGS5 polypeptide was also observed, thereby demonstrating a common binding mode for both substrates. These findings establish glycerol as a conformational activator of ADO, reveal how solvent-mediated crowding interactions can modulate substrate binding and enzymatic activity, and potentially resolve the debate regarding the nature of substrate binding at the ADO Fe-site.

## Results

### Enzymology

#### Influence of glycerol on ADO steady-state kinetics with cysteamine (CA)

In addition to NCO-activity, ADO catalyzes the O_2_-dependent formation of hypotaurine (HT) from cysteamine (CA). Therefore, ADO activity was measured by observing both the rate of O_2_-consumption and HT-formation using a calibrated Clarke-type oxygen electrode and capillary electrophoresis-mass spectrometry (CE-MS), respectively. In these experiments, the initial rate (*v*_*0*_) was normalized by the concentration of Fe(II)-containing ADO (v_0_/[E]) and values of *k*_cat_ and K_M_ were obtained from fitting the substrate dependence of v_0_/[E] to the Michaelis–Menten equation.

**Figure 2:**
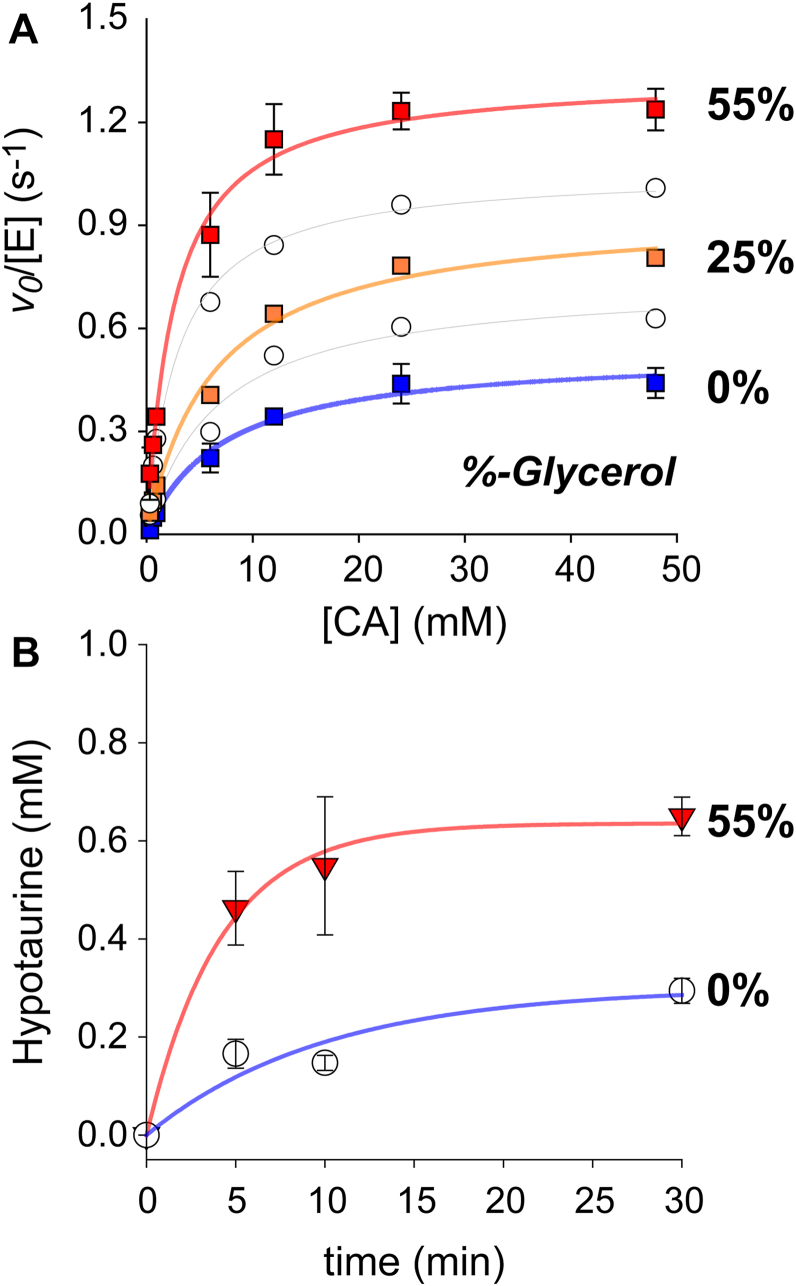
**Glycerol-dependent activation of ADO.***A*, Steady-state kinetics for ADO catalyzed reactions with CA as determined by oxygen consumption. For clarity, only error bars at the extremes (0% and 55% glycerol) are shown. Table 1 summarizes the steady-state kinetic parameters obtained from non-linear regression. *B*, CE-MS quantified initial rate of ADO catalyzed HT-formation in the absence (○) and presence (▼) of 55% glycerol.

It was previously observed by MCD and EPR that the commonly used cryoprotectant, glycerol, alters the binding of thiol-substrates at the Fe(III)-ADO active site (40). While the ferric enzyme is catalytically inert, an obvious question is whether glycerol has any impact on enzymatic activity. Figure 2 (panel **A**, *blue trace*) illustrates the steady-state curve for ADO as measured by oxygen consumption in the absence of glycerol. Fitting this data to the Michaelis-Menten equation provides a value for *k*_cat_ (0.53 ± 0.03 s^-1^) and K_M_ (7.1 ± 1.3 mM) and a resulting catalytic efficiency (*k*_cat_/K_M_) of 75 ± 14 M^-1^s^-1^. These values are largely consistent across multiple enzyme preparations. Since the reported MCD studies were performed in 55% glycerol, steady-state curves for ADO-catalyzed reactions were performed at an equivalent glycerol concentration for comparison. As shown in Figure 2*A* (*red trace*), reactions performed in 55% glycerol exhibit a near 3-fold increase in *k*_cat_ (1.34 ± 0.04 s^-1^) with a comparable decrease in K_M_ (2.6 ± 0.3 mM). This translates to a ∼7-fold increase in catalytic efficiency (520 ± 68 M^-1^s^-1^). Additional curves collected at selected glycerol concentrations [15, 25, and 40%] revealed a direct correlation between the concentration of glycerol and enzymatic activity (Fig. 2*A*). A summary of steady-state kinetic parameters obtained at each glycerol concentration is provided in Table 1.

**Table 1:** Steady-state kinetic parameters obtained at increasing glycerol content

Glycerol (%)	*k*_cat_ (s^-1^)	K_M_ (mM)	*k*_cat_/K_M_ (M^-1^s^-1^)
0	0.53 ± 0.03	7.1 ± 1.3	75 ± 14
5	0.58 ± 0.03	6.6 ± 1.0	88 ± 14
15	0.74 ± 0.05	6.5 ± 1.5	114 ± 28
25	0.94 ± 0.05	6.3 ± 1.2	150 ± 28
40	1.06 ± 0.02	2.9 ± 0.3	361 ± 34
55	1.34 ± 0.04	2.6 ± 0.3	520 ± 68

Reactions were performed in 20 mM HEPES, 50 mM NaCl, pH 8 and 25 °C.

Errors in kinetic parameters (*k*_cat_ and K_M_) were obtained from non-linear regression of the initial velocity (*v*_*0*_/[E]) with increasing substate concentration.

Among dioxygenases, the efficiency by which the enzyme incorporates molecular oxygen into its organic product is referred to as coupling. As solvent effects can influence coupling, it is possible that the increased rate of oxygen consumption observed at high glycerol concentration could reflect an uncoupling of the dioxygenase reaction, resulting in unproductive formation of reactive oxygen species and disulfides without HT formation. To evaluate this possibility, ADO-catalyzed reactions with CA were also monitored by CE-MS to directly observe product formation. Buffer excipients such as glycerol can alter the ionization efficiency of HT. Therefore, calibration curves were prepared both in the absence and presence of 55% glycerol for analytical determination of product formation from the extracted ion electropherogram of HT (Supporting Information, Fig. S1). As shown in Figure 2*B* (*blue trace*), reactions performed in the absence of glycerol exhibit time-dependent formation of HT as verified by its parent ion (110.027 m/z) and matching intensity of protonated ions (111.03 and 112.02 m/z) within the observed and simulated mass spectrum (Fig. S1*A*). Crucially, the initial rate of HT formation increased by 5-fold in 55% glycerol relative to the rate observed in its absence. Within error, the increased rate of HT-formation matches what is described above for O_2_-consumption. Therefore, the observed glycerol-dependent enhancement of ADO activity cannot be attributed to uncoupled catalysis.

#### Solvent kinetic viscosity effects

**Figure 3:**
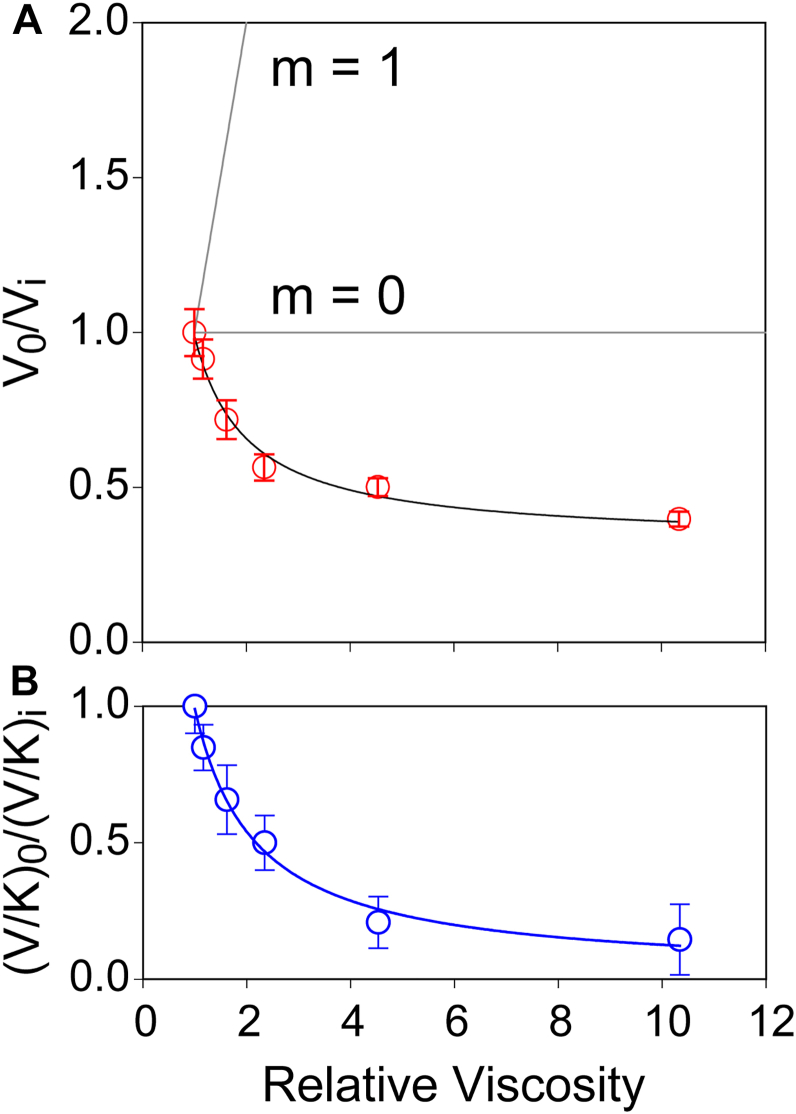
**Solvent viscosity kinetic effects on the maximal rate of ADO catalyzed HT formation.***A*, the effect of solvent viscosity on *k*_*cat*_ (*V*_*0*_/*V*_*n*_) (*A*) and *k*_*cat*_/*K*_*M*_ [(*V*/*K*)_*0*_/(*V*/*K*)_*n*_] (*B*) for oxygen consumption. The solid lines gray lines shown in *panelA* represent the theoretical limits for diffusion-limited product release.

The observed increase in both maximum velocity and catalytic efficiency for ADO is counterintuitive given that solvent viscosity is directly proportional to glycerol concentration. Consequently, diffusion-controlled events such as substrate and/or product movement into and out of the active site are expected to decrease the rate of enzymatic turnover proportionately with increasing viscosity. As described in *Materials and Methods*, kinetic parameters *k*_cat_ and *k*_cat_/K_M_ plotted as a function of relative viscosity were fit to Equation 1. For each measurement, the rate obtained in the absence of a viscogen (*v*_0_) was normalized by the value obtained at each relative viscosity (*v*_n_). Therefore, in the absence of a solvent viscosity effect, the ratio of (*v*_0_/*v*_n_) remains unaltered by increasing viscosity. Conversely, for reactions entirely limited by diffusion, a linear deflection with a unitary positive slope is expected. However, as observed in Figure 3, plots of *k*_cat_ (*panel* **A**) and *k*_cat_/K_M_ (*panel* **B**) *versus* relative viscosity exhibit an inverse rectangular hyperbolic curve, analogous to saturation kinetics. Similar nonlinear behavior has been reported previously and is attributed to an internal equilibrium or isomerization within a downstream enzyme complex that is altered in the presence of viscogen (45, 46, 47). As the inverse hyperbolic effect is observed for both *k*_cat_ and *k*_cat_/K_M_, it suggests that this activating isomerization affects the enzyme-substrate complex rather than the enzyme-product complex (47). This implies that the glycerol-dependent conformational change first identified in spectroscopic studies on the catalytically inactive ferric enzyme may also influence the rate of turnover and enzymatic efficiency in the active ferrous enzyme. Significantly, this effect is not observed in experiments using PEG-200 or sucrose as a viscogen (Fig. S2*A*). Moreover, potential contaminants (glyceraldehyde), proteins (BSA), and osmolytes (taurine) were also examined for their effect on ADO activity; however, except for glycerol, all other excipients tested inhibited ADO (Fig. S2*B*). This suggests that molecular interactions specific to glycerol, and unrelated to solvent viscosity, are responsible for this rate enhancement.

### Spectroscopy

#### Circular dichroism spectroscopy

The glycerol-dependent activation of (*k*_cat_) and catalytic efficiency (*k*_cat_/K_M_) identified in SKVE experiments described above could be the result of a protein conformational change. To examine this possibility, samples of ADO were prepared at increasing glycerol concentration [0%, 5%, 25%, and 55%] and examined by CD spectroscopy to monitor changes in protein secondary structure. As shown in Fig. S3, the observed change in CD spectra within the UV range of 195 to 255 nm is unremarkable. Secondary structure analysis suggests a 4 to 9% (Table S1) increase in α-helical content with added glycerol, which is typical for globular proteins (50). However, the change is within the error of secondary structure analysis and does not parallel the trend observed for either *k*_cat_ or *k*_cat_/K_M_. This suggests that the glycerol-dependent activation of ADO cannot be attributed to global changes in protein secondary structure.

#### Mössbauer spectroscopy

An additional concern is that glycerol may have an unanticipated impact on the first coordination sphere of the ADO Fe-site. Therefore ^57^Fe-Mössbauer spectroscopy was collected for both the resting ferrous enzyme and the enzyme-substrate complex in the presence and absence of glycerol. Figure S4 (*top*) shows the spectra collected for samples of ADO prepared in 0% and 55% glycerol. For the resting, Fe(II)-ADO enzyme prepared in the absence of glycerol, the observed zero-field Mössbauer spectrum exhibits two partially resolved doublets: δ_1_ = 1.28 mm/s, ΔE_Q1_ = 2.89 mm/s (*red*) from 75% of the iron and δ_2_ = 1.28 mm/s; ΔE_Q2_ = 2.21 mm/s (*blue*) from 25%. Both doublets were fit to a line width (Γ = 0.4) which is typical of ferrous iron. Given the large difference in quadrupole splitting (0.68 mm/s) observed for δ_1_ and δ_2_, this speciation likely reflects a difference in Fe-coordination number or charge at the Fe-site. Presumably an equilibrium between 5- and 6-coordinate as observed for both CDO and MDO (21, 51). Figure S4 (*bottom*) shows the change in the Mössbauer spectrum upon addition of 20 M equivalent CA per ADO. Here, both doublets observed in the free enzyme exhibit a decrease in the isomer shift (Δδ = −0.06 mm/s) [δ_3_ = 1.22 mm/s; ΔE_Q__3_ = 2.86 mm/s; δ_4_ = 1.22 mm/s; ΔE_Q__4_ = 2.21]. While smaller than observed for CDO (10) or MDO (28) (Table S2), the decrease in both δ_1_ and δ_2_ suggests that both Fe-sites bind CA *via* direct S-atom coordination. As both doublets are shifted in the presence of substrate, the observation of Fe-site speciation most likely represents heterogeneity within the ADO Fe-site rather than contributions from adventitious ferrous iron. Of note, the relative distribution of each doublet [76:24] is largely unaltered in the presence of substrate. The observed Mössbauer parameters (δ and ΔE_Q_), relative distribution of doublets, and magnitude of decrease in δ upon addition of CA are all consistent with values previously reported for ADO (41).

As shown in Table S2, the Mössbauer parameters obtained for resting Fe(II)-ADO in 55% glycerol are nearly equivalent to samples prepared in the absence of glycerol [δ_1’_ = 1.27 mm/s, ΔE_Q1’_ = 2.91 mm/s and δ_2’_ = 1.27 mm/s; ΔE_Q2_’ = 2.21 mm/s]. Of note, the relative distribution observed for each doublet changes slightly, with 83% δ_1_ and 17% δ_2_. Equivalent glycerol samples (55%) in which 20-fold CA is added relative to Fe(II)-ADO again show the same decrease in the isomer shift (Δδ = −0.06 mm/s) for both doublets [δ_3__’_ = 1.21 mm/s, ΔE_Q__3__’_ = 2.86 mm/s and δ_4__’_ = 1.21 mm/s; ΔE_Q__4__’_ = 2.21 mm/s] with no significant perturbation to the distribution of iron sites [85:15].

#### EPR spectroscopy

Despite the enhanced activity of ADO observed in the presence of glycerol (*vide supra*), no obvious perturbations in protein secondary structure are observed. This suggests that glycerol may have a more localized impact on the enzyme-substrate complex which favors catalytic turnover. Therefore, EPR spectroscopic investigations previously reported for ADO merit reexamination. In the first set of experiments, we intend to replicate the conditions in which glycerol-dependent conformational change is observed in the ferric enzyme. Once this is accomplished, experiments using nitric oxide will be used as a spectroscopic probe to interrogate the substrate-binding denticity of the enzyme-substrate-NO ternary complex.

**Figure 4:**
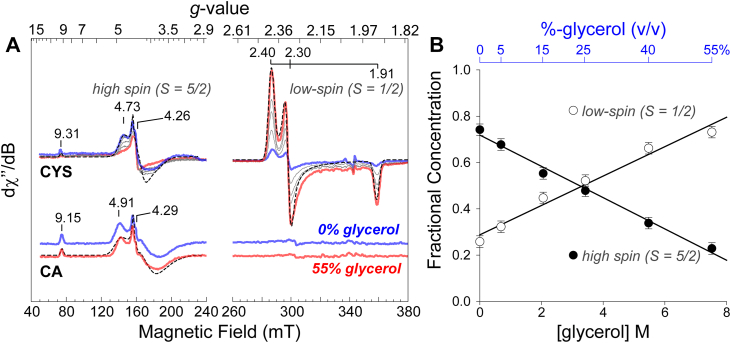
**Effect of glycerol on CYS and CA binding to the oxidied Fe(III)-ADO.***A*, 10 K CW EPR spectra of CYS- (*top*) and CA-bound Fe(III)-ADO (*bottom*) with increasing glycerol concentration. Selected glycerol concentrations are varied from 0% [*blue*], 5%, 15%, 25%, 40%, and 55% [*red*] *Instrumental parameters*: microwave frequency, 9.63 GHz; microwave power, −30 dB (0.2 mW); modulation amplitude, 0.9 mT; temperature, 10 K. *B*, quantitation of high- and low-spin CYS-bound Fe(III)-ADO EPR species with increasing glycerol concentration. Quantitation of high-spin CYS/CA-bound Fe(III)-ADO was performed by analytical simulation of EPR spectra. Table S3 summarizes the EPR simulation parameters obtained for each complex as well as experimentally determined values for axial zero field splitting terms (*D*) (Fig. S5).

Figure 4 (panel A) shows the EPR spectra of Fe(III)-ADO (0.3 mM) in the presence of a 10-fold molar excess CYS (top) and CA (bottom). While deviations in the observed *g*-values indicate minor differences in zero field splitting terms [*D* and *E*/*D*] when bound to each ligand, both signals are diagnostic of a rhombic high-spin (*S* = 5/2) ferric center. In both spectra, the central transition observed near *g* ∼ 4.3 is flanked by a shoulder at *g* ∼ 4.7 for CYS and 4.9 for CA. This signal originates from a transition within the middle doublet (levels 3-4) of the *S* = 5/2 spin system near the rhombic limit (*E*/*D* ∼ 0.33). The weaker transition observed at a *g*-value of 9.3 for CYS and 9.1 for CA is attributed to a transition within the ground doublet (levels 1-2).

The energy separating the ground and first excited state (2*D*) is defined by the axial zero-field splitting term, *D*-value. This value was experimentally measured for both CYS- and CA-Fe(III)-ADO by measuring the temperature-normalized signal area (S × T) for the ground and first excited state signals and plotting this data as a function of temperature. The data from each doublet were then fit to a Boltzmann population curve for a 3-level system using Equation S1 (Fig. S5, *A* and *B*). From this analysis, it was observed that the *D*-value for CYS and CA-Fe(III)-ADO are closely matched (2.2 ± 0.3 and 1.6 ± 0.3 cm^-1^, respectively). Furthermore, spectroscopic simulations reveal that the Fe-site is slightly more rhombic when bound to CYS (*E*/*D* = 0.25) relative to CA (*E*/*D* = 0.23). Quantitative spectroscopic simulations (*dashed lines*), which include the experimentally determined *D*-values, are overlaid on each signal for comparison. The simulations faithfully reproduce the observed line shape as well as the relative intensity of ground and middle doublet transitions. The spectroscopic parameters determined here are largely consistent with those reported previously, indicating that we are observing the same enzyme-substrate species (40). However, it should be noted that the previously reported axial zero-field splitting (*D*) values were obtained from a two-component simulation of a spectrum recorded at a single temperature and are therefore approximate. In contrast, the *D*-values reported here were derived from analytical fits of data collected over a broad temperature range (Fig. S5) and are consequently more accurate. A summary of the spectroscopic parameters used in EPR simulations is presented in Table S3.

Significantly, samples of CYS-Fe(III)-ADO prepared in the presence of glycerol show an additional signal with observed *g*-values of 2.40, 2.30, and 1.91, consistent with a low-spin (*S* = 1/2) ferric site. These *g*-values matched those reported by Fernández *et al.* in their initial EPR study (40). As shown in Figure 4 (panel B), the amount of high-spin CYS-Fe(III)-ADO decreases concomitant with the formation of this new low-spin signal as the concentration of glycerol increases. By contrast, when the same experiment was performed for the samples of CA-Fe(III)-ADO, no change was observed in the amount of high-spin iron present. Further, no low-spin iron signals are observed whatsoever. The results reported here are entirely consistent with the initial EPR study and verify that the binding denticity of CYS (and not CA) can be altered from monodentate to bidentate by the addition of glycerol. As the conditions favoring a glycerol-dependent conformational change have been reproduced, our next step is to examine how glycerol alters substrate binding in the catalytically active, Fe(II)-ADO.

The above mentioned EPR suggests that only the binding denticity of CYS was influenced by glycerol, whereas the binding of the native CA-substrate remained unaffected. However, steady-state assays and SKVE studies (*vide supra*) reveal a glycerol-dependent enhancement in both *k*_cat_ and *k*_cat_/K_M_ for Fe(II)-ADO. As substrate coordination is strongly influenced by the metal oxidation state (52), it is possible that spectroscopic studies performed on the ferric enzyme do not accurately reflect perturbations within the catalytically active ferrous enzyme-substrate complex. To explore this possibility, EPR experiments were performed on catalytically active Fe(II)-ADO using nitric oxide (NO) as a spectroscopic probe for dioxygen binding. The resulting (CA/NO)-ADO ternary complex is structurally analogous to the putative iron-superoxo enzyme substrate-complex reported elsewhere (53). While similar experiments were reported previously, the influence of glycerol was not examined in that work. Further, key control experiments and spin-quantitation were omitted, which may have contributed to the misinterpretation of results (41) (See Figs. S6-S8).

**Figure 5:**
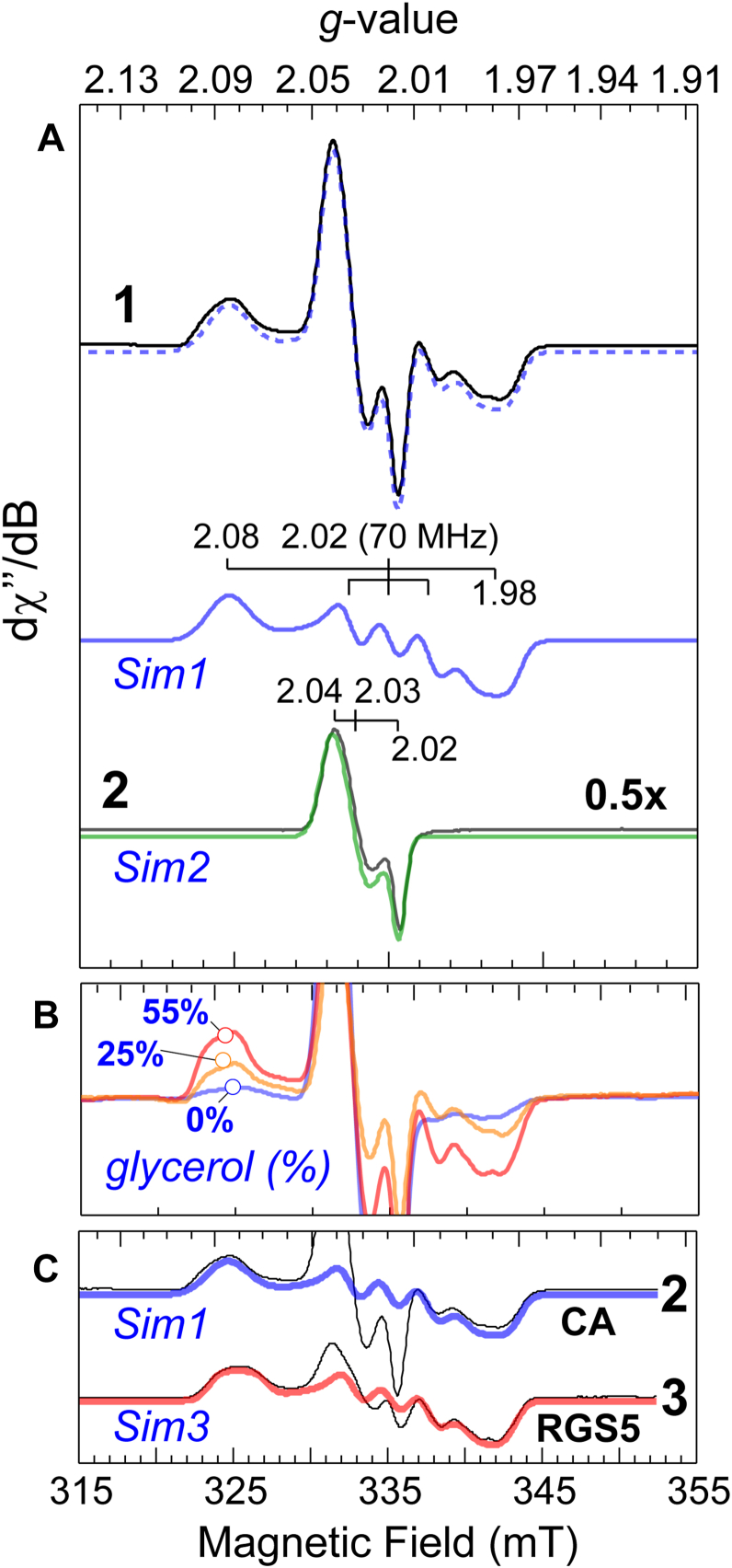
**Effect of glycerol on ADO substrate-bound iron-nitrosyl formation.***A*, trace 1 shows the observed CW X-band EPR spectrum collected at 10 K for Fe(II)-ADO pre-incubated with CA in 55% glycerol following treatment with NO. The two-component simulation (*dashed line*) is comprised of two species idicated by *Sim1* and *Sim2* (*blue lines*). Trace 2 shows the CW EPR spectra observed upon addition of NO to an anaerobic solution of Fe(II) and CA in the absence of ADO. *B*, CW EPR spectra illustrating the increase in (CA/NO)-bound ADO observed with increasing glycerol concentration. *C*, presents the EPR spectra observed for (RGS5/NO)-bound ADO when the polypeptide substrate is substituted for CA. *Instrumental parameters*: microwave frequency, 9.47 GHz; microwave power, −40 dB (21 μW); modulation amplitude, 0.9 mT; temperature, 10 K.

Samples of Fe(II)-ADO (0.3 mM) were prepared in anaerobic buffer with selected concentrations of glycerol and allowed to pre-incubate with 10-fold molar equivalents of CA prior to the addition of 10-fold molar equivalents of NO. Figure 5*A* (*trace* **1**) shows the EPR spectrum for CA-Fe(II)-ADO treated with NO in the presence of 55% glycerol. This signal can be simulated assuming two spectroscopically distinct (*S* = 1/2) species (*dashed line*) and decomposed into the individual contributions from each species, indicated by *Sim 1* and *Sim2*. Table 2 summarizes the EPR spectroscopic parameters used to simulate each (*S* = 1/2) iron-nitrosyl species.

**Table 2:** EPR simulation parameters used for iron-nitrosyl ADO species and adventitious dinitrosyl iron complex (DNIC)

Sample	Spin	*g*_x_	*g*_*y*_	*g*_*z*_	*A*_x_	*A*_y_	*A*_z_	σ_B_ (mT)
(CA/NO)-ADO	*S* = 1/2	2.086	2.018	1.982	30	74	34	0.5
(RGS5/NO)-ADO	*S* = 1/2	2.081	2.019	1.981	37	68	30	0.5
CA-DNIC	*S* = 1/2	2.044	2.033	2.016	-	-	-	0.5

*Sim1*(*blue*) exhibits *g*-values of 2.086, 2.018, and 1.982 and a central triplet hyperfine (*A* = 74 MHz) splitting associated with strong-coupling to a single nitrogen atom (*I* = 1). This signal is reminiscent of CDO simultaneously coordinated by CYS and nitric oxide (CYS/NO)-CDO. For CDO, this signal has been assigned to a low-spin iron-nitrosyl produced by bidentate coordination of CYS to the CDO Fe-site *via* thiol- and neutral amine (31, 42, 54). Given the functional and structural similarities between CDO and ADO, it is reasonable to conclude that the iron-nitrosyl EPR signal observed for ADO also reflects bidentate coordination of CA to the ADO Fe-site.

Significantly, Figure 5 (*panel* B) demonstrates that the amount of (CA/NO)-ADO observed by EPR is directly proportional to the amount of glycerol present in solution. At 55% glycerol, the fraction of (CA/NO)-ADO accounts for 80% of the observed iron. However, the concentration of this species decreases by 4-fold in samples prepared in the absence of glycerol. Thus, despite the absence of an observable effect with the oxidized enzyme (Fig. 4), glycerol does indeed alter the binding denticity of CA to favor bidentate coordination to the catalytically relevant ferrous iron site. As the fraction of (CA/NO)-ADO iron-nitrosyl species, enzymatic turnover, and catalytic efficiency all scale with added glycerol content, we interpret this result to mean that the catalytically relevant, O_2_-activating enzyme-substrate complex, exhibits bidentate coordination of substrate *via* thiolate and amine functional groups, as verified for CDO.

The second species revealed by *Sim2 (green)*, exhibits *g*-values of 2.04, 2.03, and 2.02 and can be attributed to a known four-coordinate dinitrosyl iron complex (DNIC) produced by adventitious iron, free ligand, and NO (55, 56). From the two-component simulation, it was determined that the DNIC associated with *Sim2* accounts for ∼20% of the total iron observed. Following the published stoichiometry for DNIC synthesis [3 NO: 1 Fe(II): 2 CA] (57, 58), this signal can be exactly reproduced in the protein buffer (Fig. 5*A*, *trace* 2) by anaerobic addition of NO to [Fe(NH_3_)_2_(SO_4_)_2_] in the presence of CA. In addition to matching *g*-values, the control sample of DNIC also exhibits equivalent relaxation properties as reflected by the microwave power required for half-saturation (Fig. S7 and Table S4). Crucially, as this signal is produced in the absence of ADO, it cannot be attributed to any enzyme- or enzyme-substrate iron nitrosyl complex as reported previously (41). Instead, the observed DNIC appears to be the result of promiscuous reactivity of NO with adventitious ferrous iron and free CA ligand. This conclusion is also supported by quantitative EPR experiments in which titration of the NO-bound ADO with excess CA was performed (Figs. S6 and S8). Unfortunately, the omission of this control experiment likely contributed to the misassignment of this species in the initial study (41). As the species represented by *Sim2* is unrelated to substrate-binding at the ADO Fe-site, it will not be discussed further.

The above experiments reveal a correlation between glycerol content and bidentate substrate coordination of CA. An obvious question is how these iron-nitrosyl signals compare when bound to an N_t_-CYS residue. To address this question, samples of ADO were pre-incubated with a synthetic 14-amino acid polypeptide (CKGLAALPHSCLER) corresponding to the N-terminal sequence of a G-protein regulator of protein stability (RGS5) (41). Samples of lyophilized RGS5 polypeptide were reconstituted and then introduced ADO anaerobically to yield a final concentration of ADO (0.3 mM) and RGS5 (2.5 mM) in the absence of glycerol. The mixture was then allowed to equilibrate on ice for 10 min prior to the addition of 10-fold molar equivalents of NO. As shown in Figure 5*C*, samples of (RGS5/NO)-ADO exhibit *g*-values of 2.081, 2.019, 1.981 and a central triplet hyperfine splitting of 68 MHz. These features are nearly identical to the low-spin iron nitrosyl observed in samples of (CA/NO)-ADO. Significantly, no (*S* = 3/2) signals are observed by EPR, which would indicate binding of NO to ADO in the absence of substrate, or an equilibrium between mono- and bidentate N_t_-CYS-coordination.

Analytical simulation of this signal accounts for 80% of the total iron in the sample, indicating that the bulk of the enzyme coordinates RGS5 bidentate in the absence of glycerol. Comparison to samples prepared in the presence of 55% glycerol shows a slight increase (∼15%) in the amount of low-spin (RGS5/NO)-ADO iron-nitrosyl observed, but no other perturbation to the EPR signal. This result confirms that N_t_-CYS-residues bind to the ADO bidentate and its binding is not significantly influenced by glycerol content.

Continuous wave EPR experiments discussed above reveal significant molecular rearrangement of the Fe-site first coordination sphere. Since glycerol does not directly coordinate with or alter the Fe-site (Fig. S4), the shift from monodentate to bidentate CA-coordination and resulting rate enhancement for ADO is likely attributed to crowding effects within the active site outer coordination sphere. Multiple crystal structures of resting ADO reveal a common binding site for glycerol experimentally introduced as a cryoprotectant (36, 53). In these structures (Fig. S9), glycerol binds within the substrate binding channel and within 5.0 Å of the enzymatic Fe-site. If binding of glycerol at this position persists in solution, it could easily explain how glycerol influences CA-binding. To corroborate this conclusion, pulsed EPR experiments (3-pulse Electron Spin Echo Envelope Modulation, 3p ESEEM) were performed on samples of (CA/NO)-ADO. In this study, samples prepared in 55% d_8_-glycerol were compared to those prepared in natural abundance glycerol.

**Figure 6:**
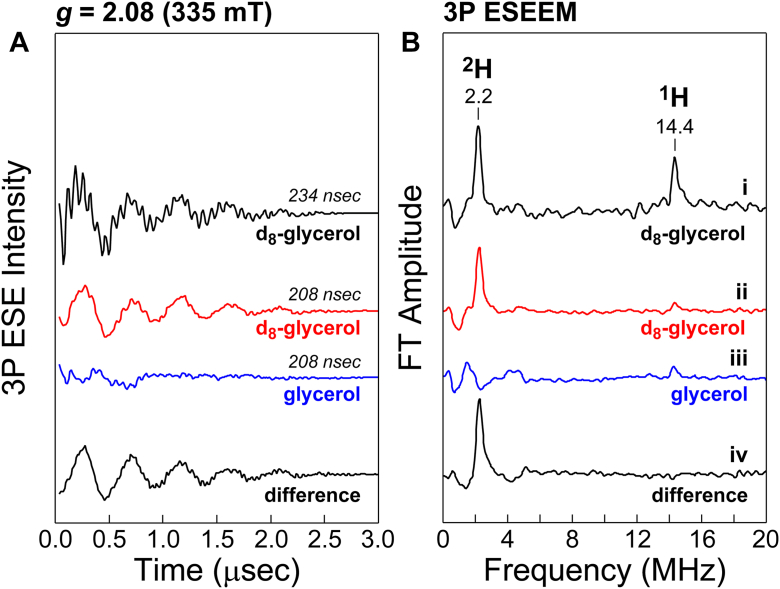
**Pulsed EPR spectroscopy of ADO substrate-bound iron-nitrosyl.** Three pulse ESEEM spectra (4 K) collected for Fe(II)-ADO pre-incubated with CA in 55% glycerol (*blue*) and d_8_-glycerol (*red*) following treatment with NO. The time (*A*) and frequency (*B*) domian of the 3P ESEEM experiment is presented separately for ease of comparison. Values of tau (234 and 208 nsec) were selected for maximal and minimal proton modulation, respectively. For ease of comparison, the time-dependent spectra were phased to minimize the imaginary component, then the natural log was taken, prior to subtracting off a second-order polynomial baseline. A sine bell apodization function was applied to minimize noise. The result was zero-filled to 2048 points, and the Fourier transform was calculated (*panelB*). *Instrumental parameters*: microwave frequency, 9.78 GHz; microwave power, 5 dB; shot repetition, 15,000 μsec.

The maximum distance for observing weakly coupled magnetic nuclei to a paramagnetic center using ESEEM is < 8 Å (59, 60). Accordingly, only ^2^H nuclei located within the outer coordination sphere of the paramagnetic center are expected to produce periodic modulation of the electron spin echo as a function of time. Therefore, ^2^H nuclei present in the bulk solvent should not be observed. Figure 6*A* shows the time-domain data from a 3-pulse ESEEM experiment collected at 335 mT for (CA/NO)-ADO prepared in 55% d_8_-glycerol. A delay time (τ = 234 ns) was chosen to maximize the 3-pulse modulation intensity for both ^1^H and ^2^H nuclei. At 9.78 GHz, this field corresponds to *g* = 2.08 in the CW EPR spectrum (Fig. 5). The 3p ESEEM spectrum exhibits oscillations at ∼70 ns and 455 ns, consistent with couplings to ^1^H and ^2^H nuclei, respectively. Fourier transformation of the time-domain data (Fig. 6*B*, trace ***i***) yields the corresponding frequency-domain spectrum, which corroborates this assignment by matching the expected ENDOR frequencies of 14.4 MHz (^1^H) and 2.2 MHz (^2^H) at this field position.

Measurements were also made using a shorter delay time (τ = 208 ns) to minimize contributions from coupled protons. This adjustment nearly eliminated ^1^H modulation in both the time- and frequency-domain spectrum (trace ***ii***). Additional comparison with samples prepared in 55% natural-abundance glycerol demonstrates that the long-lived, low-frequency ^2^H modulation is abolished (trace ***iii***). Instead, multiple broad peaks below 5 MHz are observed, which originate from weak ^14^N nuclei (*I* = 1) near the iron–nitrosyl center. This modulation can be attributed to the three Fe-coordinated histidine residues in the active site. To remove these contributions from the data, a “difference” spectrum (^2^H-^1^H) was obtained by subtraction of the normalized natural-abundance time-domain spectrum from the d_8_-glycerol data prior to Fourier transformation. The resulting frequency domain spectrum is shown Figure 6*B* (trace ***iv***). Pulsed EPR experiments confirm the presence of glycerol within the outer-coordination sphere of the (CA/NO)-ADO site and can therefore directly influence substrate-binding by molecular crowding.

### Computational modeling

#### Optimized structures of the (CA/NO)-ADO ternary complex

Steady-state assays performed for ADO demonstrate increased catalytic turnover with increasing glycerol content. Complementary EPR studies performed on the catalytically active Fe(II)-ADO demonstrate that glycerol increases the fraction of bidentate CA-coordination within the substrate-bound iron nitrosyl complex. Taken together, these studies suggest that the catalytically relevant binding mode for CA is bidentate rather than monodentate. To examine this further, DFT computational modeling was used to determine the lowest energy bidentate binding conformation for CA.

**Figure 7:**
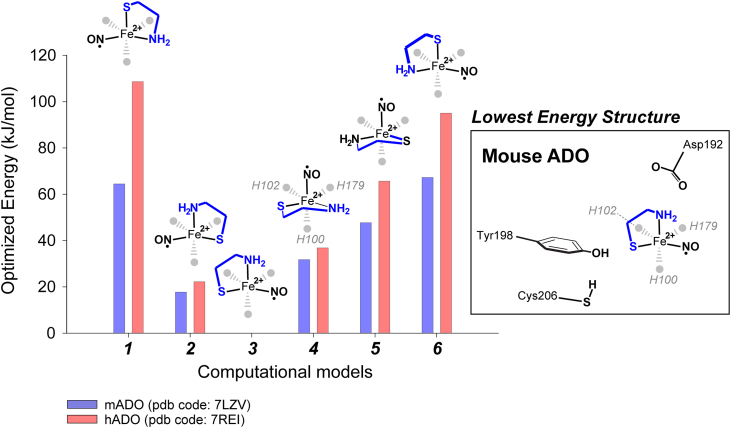
**Normalized energies for all possible CA-binding conformations in optimized ADO models.** Inset illustrates all first- and outer-sphere coordination interactions accounted for in DFT optimized models.

Two sets of models were generated using the starting crystallographic coordinates for the mouse (PDB code 7LVZ) and human (PDB code 7REI) ADO crystal structure (35, 36). A comparison of selected bond distances and angles for each Fe-site is provided in Table S5. For ease of comparison, the numerical designation of active site amino acids used the Mouse ADO sequence. Both structures included the central iron, atoms from the three-histidine facial triad [His100, His102, and His179], and the three closest outer-sphere residues [Asp192, Tyr198, and Cys206]. Nitric oxide was added as the sixth ligand to mimic the structure of the iron-nitrosyl characterized by EPR. The structures were further edited by capping the alpha carbons for each residue with methyl groups. The six different bidentate binding poses were generated for both mouse and human ADO, and the relative energies were compared to determine the lowest energy configuration. Figure 7 summarizes the energies calculated for each CA-binding mode within the ADO active site. Significantly, the trend in relative energies for each structure are largely consistent regardless of the XRD structure used as starting coordinates.

The two highest energy conformations [**1**] and [**6**] place the CA-thiolate near (3.4 Å) the outer-sphere Asp192 residue. These also exhibit the greatest divergence, separating their normalized optimized energy. This is expected given the proximity of the negatively charged thiolate S-atom (Mulliken atomic charge −0.6) and the nearest O-atom of the Asp192 carboxylate (Mulliken atomic charge, −0.4).

Similarly, replacement of the substrate thiolate at the axial Fe-position with NO yields the slightly more stable conformers [**4**] and [**5**], and greater agreement among optimized energies. The electronic structure of the (CA/NO)-ADO (*vide infra*) can be treated as a low-spin ferrous iron coordinated to a ligand-centered radical. However, despite the neutral character of the Fe-bound NO, the distal O-atom of the Fe-bound NO has some anionic character (Mulliken atomic charge, −0.2) and thus its placement near (3.1–3.5 Å) the carboxylate of Asp192 is unstable.

When considering the energetic trend in substrate-binding ([**1**] > [**6**] > [**5**] > [**4**]), it appears that placement of any negative charge near the carboxylate group of Asp192 is energetically uphill. Recent work by Li *et al.*, identified a Co(III)-superoxide intermediate as the substrate-oxidating species for Co(II)-substituted ADO (53). If this observation is extrapolated to the native Fe(II)-ADO, it can be assumed that iron(III)-superoxide is a catalytically relevant intermediate. Since reductive activation of dioxygen is expected to accumulate a higher anionic charge relative to NO, it’s difficult to imagine how this position could represent an O_2_-binding site. To test this hypothesis, optimized structures were generated for a putative iron(III)-superoxide intermediate in substrate-binding conformation [**3**] and [**4**] to compare their relative energies. As expected, both proximal and distal O-atoms of the Fe(III)-bound superoxide exhibit greater anionic character relative to NO (Mulliken atomic charges, proximal −0.16; distal, −0.24), translating to a much larger energy difference separating these models. This suggests that binding of dioxygen to the iron coordination site *trans* to His100, and adjacent to Asp192, is unfavorable.

The two lowest energy conformers, [**2**] and [**3**], share a similar feature in that the substrate-amine is positioned closest in proximity (3.1 Å) to the active site aspartate. Significantly, PCO and ADO variants at the equivalent Asp192 residue show abolished (or minimal) activity, indicating that this residue is catalytically essential (37, 38, 39). This suggests that Asp192 plays a crucial role in facilitating substrate coordination at the ADO Fe-site. For instance, Asp192 may function as a proton acceptor for the CA- (or N_t_-CYS) alpha amine. This would facilitate Fe-coordination of the neutral amine while simultaneously neutralizing the charge on both Asp192 and the substrate amine.

For both mouse and human ADO structures, the lowest energy conformation, [**3**], positions the CA S-atom within hydrogen bonding distance (2.1 Å) of Tyr198. As all structures with the S-atom positioned further from Tyr198 are higher in energy, it is reasonable to conclude that H-bond donation from Tyr198 stabilizes the anionic S-atom of the Fe-bound thiolate.

**Figure 8:**
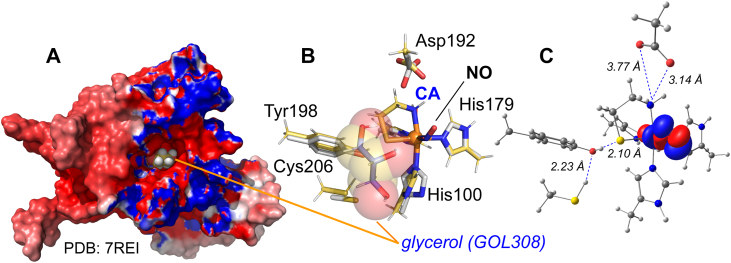
**Optimized model of glycerol binding site and electronic structure of (CA/NO)-ADO.***Panel A* shows a surface plot of the ADO XRD structure (PDB: 7REI). The structurally conserved cryoprotectant (GOL308) capping the substrate binding channel is shown as a space fill model. *Panel B* overlays GOL308 with the geometry optimized structure of ADO (conformation 3). This structure illustrates how molecular crowding from glycerol may force bidentate CA-coordination at the ADO active site. *PanelC* shows an isosurface plot (contour value, 0.05) for the singly occupied molecular orbital (SOMO) of the low-spin (CA/NO)-ADO iron-nitrosyl site.

As discussed above, multiple crystal structures of resting ADO reveal a common glycerol binding site near the ADO Fe-site. Figure 8*A* illustrates how the placement of GOL308 (*spheres*) in the human ADO (PDB 7REI) caps the substrate-binding channel. Crucially, the positioning of this GOL308 packs against the optimized Fe-bound CA of the DFT optimized conformation [**3**] without any conflicts in van der Waals radii (Fig. 8*B*).

#### Electronic structure of the (CA/NO)-ADO

EPR spectroscopic parameters (*g*- and *A*-tensors) were calculated by DFT to provide additional corroboration of models (Table S6). Calculations were performed using multiple functionals (BP86, B3LYP, and TPSSH); however, greatest overall agreement with spectroscopic values was obtained using the generalized gradient approximation (GGA) functional, BP86. While all models predict a relative *g*-anisotropy consistent with experimental results, the magnitude of the span (*g*_max_ – *g*_min_) is underestimated regardless of the functional used. However, the calculated *A*-values faithfully reproduce the large central N-atom hyperfine splitting consistent with a ligand-centered NO-radical. To gain further insight into the nature of the (CA/NO-ADO electronic structure, the molecular orbitals were calculated. Figure 8*C* illustrates the singly occupied molecular orbital (SOMO) calculated for conformation [**3**]. An isosurface plot of this orbital illustrates that the bulk of the unpaired electron density is localized within the *π∗* orbital of the coordinated NO ligand. However, additional mixing from the iron *d*_π_-orbital likely contributes to the observed *g*-anisotropy of the EPR spectrum. These features are consistent with those reported for (CYS/NO)-CDO and synthetic model complexes of this enzyme (61, 62, 63).

## Discussion

### Glycerol-dependent ADO activation

The work presented here shows that glycerol significantly enhances catalytic activity and efficiency for ADO by promoting bidentate coordination of cysteamine (CA) at the ferrous iron site. Steady-state kinetic analyses revealed a near 7-fold increase in *k*_cat_/K_M_ at high glycerol concentration (55%), while alternative viscogens (PEG-200, sucrose) show vastly attenuated rates of catalysis. By contrast, the observed solvent viscosity effects for these viscogens (Fig. S2) imply a more complicated interplay between solvent viscosity effects and changes in the local protein dynamics that contribute to catalysis (64, 65). Conversely, the inverse hyperbolic dependence of activity observed for glycerol in SKVE studies indicates that the rate enhancement arises from conformational activation rather than diffusion-limited processes. This glycerol-dependent activation parallels a structural perturbation first reported in spectroscopic studies on the oxidized Fe(III)-ADO, although the effect on catalysis is only evident in the catalytically active ferrous state. Additional control experiments performed with glyceraldehyde, BSA, and taurine, did not produce comparable activation of ADO. Indeed, among all excipients examined (Fig. S2), conformational activation was only observed for glycerol.

### Spectroscopy of Fe(III)- and Fe(II)-ADO

To identify the structural basis for the glycerol-dependent rate enhancement, several spectroscopic techniques were applied to the characterization of ADO. Circular dichroism experiments showed only minor changes in secondary structure, suggesting that glycerol induces local rather than global rearrangements. EPR experiments performed on the oxidized Fe(III)-ADO are largely consistent with previous reports that confirm a glycerol-dependent change in the CYS-bound ADO site from high-spin (*S* = 5/2) to low-spin (*S* = 1/2) ferric iron. Significantly, this is not observed for the catalytically relevant CA-substrate despite a clear activation of the enzyme in the active, ferrous iron oxidation state. One point worth mentioning is that it is not known if CA and CYS coordinate at equivalent positions. Since ADO exhibits no product formation in reactions with CYS, it is reasonable to assume that the active site exhibits some structural feature to distinguish these nearly equivalent molecules. For instance, electrostatic repulsion from Asp192 may play a role in destabilizing coordination of the CYS-amine group at the most stable binding conformation for CA [**3**]. Moreover, the metal oxidation state can have a considerable influence on substrate binding, and thus, additional spectroscopic measurements were performed on the catalytically active, ferrous enzyme.

Reduced ADO was initially characterized by Mössbauer spectra to verify that glycerol does not alter the first coordination sphere of either the resting enzyme or enzyme-substrate complex. These experiments confirmed that no significant changes are observed in either isomer shift (δ) or quadrupole splitting (ΔE_Q_) upon addition of glycerol. Further, the values reported are consistent with those previously reported for human Fe(II)-ADO in the resting, and substrate-bound complex (41). Finally, Mössbauer spectra established the absence of adventitious iron present in purified samples of ADO. As discussed in Supporting Information (Figs. S7 and S8), this is an important control to perform prior to experiments involving NO addition in the presence of exogenous ligands (CA) as free ferrous iron contributes to the production of dinitrosyl complexes (DNIC) (57, 58).

Irrespective of the presence of glycerol, it is curious that the Fe(II)-ADO Mössbauer spectroscopic features change only subtly following anaerobic incubation with excess CA. The ^57^Fe-Mössbauer isomer shift (δ) correlates primarily with changes in Fe 4s electron density, therefore the more electron-rich N-atom of cysteamine is expected to increase 4s population at the Fe-site through enhanced σ-donation (66, 67, 68). Consequently, a slight decrease in δ is expected upon substitution of one water ligand O-atom with the cysteamine N-atom (69). This trend is illustrated by comparison of the simple inorganic complexes [Fe(H_2_O)_6_]^2+^ (δ = 1.30 mm/s, ΔE_Q_ = 3.1–3.3 mm/s) and [Fe(NH_3_)_6_]^2+^ (δ = 1.25 mm/s, ΔE_Q_ = 2.6–3.0 mm/s), where the N-centered complex exhibits a slightly lower isomer shift (Δδ ∼ 0.05 mm/s) relative to hexaqua (68, 70). No doubt this perturbation would be much smaller upon substitution of a single O-atom donating ligand for an N-atom donor. However, among protein samples, this phenomenon is exceedingly more difficult to resolve due to their increased linewidths (Γ). The inherent structural heterogeneity (68, 71) of protein samples often results in increased linewidth as observed here for the as-isolated Fe(II)-ADO and Fe(II)-ADO:CA complex (Γ ∼ 0.4 mm/s). This makes resolving any isomer shift changes uniquely originating from the cysteamine N-atom alone challenging.

Of greater interest is the surprisingly small change in the observed isomer shift (Δδ) for Fe(II)-ADO upon coordinating to the σ-donating thiol moiety of CA. Table S2 illustrates the observed Mössbauer parameters reported for smTDO enzymes (CDO and MDO) and their corresponding enzyme-substrate complex. Both enzymes exhibit a much greater decrease in isomer shift (Δδ = −0.12–0.19 mm/s) upon substrate-coordination relative to Fe(II)-ADO (−0.06 mm/s) (21, 28, 51, 72). As this behavior is unique to ADO, it likely reflects a unique structure feature within the Fe(II)-ADO:CA complex, which influences the covalency of the Fe-S bond. Based on the structure shown in Figure 8, we speculate that this behavior is attributed to Tyr198 H-bond donation to the Fe-coordinated S-atom of CA. H-bond donation is expected to decrease the σ-donor strength of the CA thiol, thereby reducing Fe–S bond covalency. Consequently, H-bond donation should yield a more positive isomer shift relative to that observed in its absence. This reasoning is consistent with multiple literature reports in which Mössbauer spectroscopy was used to examine the influence of H-bond donation on Fe-S covalency for both heme and non-heme iron enzymes (73, 74, 75).

EPR studies performed on the resting enzyme utilized nitric oxide as a spectroscopic probe for the binding of substrate at the ADO Fe-site. Samples of (CA/NO)-ADO revealed a low-spin iron-nitrosyl signal. The observed *g*-values for (CA/NO)-bound ADO [2.08, 2.02, and 1.98] are remarkably similar to those reported for (CYS/NO)-CDO [2.07, 2.02, and 1.98] (42). Crystallographic studies performed on CDO in complex with CYS and nitric oxide (PDB 6N43) confirmed bidentate coordination of CYS at the iron center (31). Given the structural similarities between CDO and ADO, the observed spectra support the conclusion that CA is coordinated bidentate within the enzyme-substrate iron-nitrosyl ternary complex. Therefore, despite seeing no perturbation to CA-coordination in the ferric enzyme (Fig. 4), glycerol does indeed alter the binding denticity of CA to favor bidentate coordination to the catalytically relevant ferrous iron site. Further, since the fraction of (CA/NO)-ADO iron-nitrosyl species, enzymatic turnover, and catalytic efficiency all scale with added glycerol content, we interpret this result to mean that the catalytically relevant, O_2_-activating enzyme-substrate complex exhibits bidentate coordination of substrate *via* thiolate and amine functional groups as reported for CDO.

Crucially, in samples where the RGS5 peptide is substituted for CA, the observed *g*-values are nearly superimposable with those observed for (CA/NO)-ADO. This indicates that both substrates coordinate to the Fe-site in an equivalent, bidentate manner. Further, as glycerol has minimal effect on the amount of (RGS5/NO)-ADO observed, it can be inferred that bidentate substrate-coordination is preferred for N_t_-CYS-residues. Notably, a minor decrease in the *g*-largest numerical *g*-value (2.081 *versus* 2.086) was observed for samples of (RGS5/NO)-ADO relative to those prepared from CA. There is also a slight decrease in the magnitude of the central hyperfine splitting for these species (68 *versus* 74 MHz). This difference may arise from subtle changes in active site geometry caused by the larger RGS5 peptide, or from electronic perturbations within the iron–amine/thiolate bonds due to inductive effects from the adjacent amide carbonyl group. Regardless, these experiments clearly demonstrate that interactions with glycerol stabilize bidentate coordination of CA, thereby providing a structural explanation for the increased rate of catalysis observed in steady-state experiments.

Multiple crystal structures of resting ADO (PDB codes: 8UAN, 8U9J, and 7REI) revealed a common binding site for glycerol experimentally introduced as a cryoprotectant. In these structures (Fig. S9), glycerol is positioned within the substrate binding channel and lies within 5.0 Å of the enzymatic Fe-site. Figure 8*B* shows the alignment of the 7REI structure with the DFT-optimized model, conformation [**3**]. Significantly, no conflicts in van der Waals radii are observed between glycerol and Fe-bound CA. From this structure, it is easy to imagine how molecular crowding from glycerol could stabilize bidentate CA-coordination at the Fe-site. While pulsed EPR (ESEEM) experiments cannot verify that glycerol binds to the same position as GOL308 (Fig. 8*B*) in solution, it does verify the presence of d^8^-glycerol within the outer coordination sphere of the Fe-site since only deuterium nuclei within ∼8 Å of the iron-nitrosyl can be observed.

### Structure of the (CA/NO)-ADO ternary complex

The lowest energy bidentate CA conformation positions the substrate thiolate within hydrogen-bonding distance of Tyr198 and the amine near Asp192. Of note, this substrate-binding conformation [**3**] deviates from the structure of Co(II)-substituted ADO in complex with CP6-LK8-Ser (Fig. 1*C*), which would be most consistent with conformation [**4**]. While the CP6-LK8-Ser peptide is a potent inhibitor of ADO activity, it exhibits considerable deviation in both sequence, structure, and charge as compared to native N-terminal sequence of target RGS proteins.

Moreover, this cyclic peptide is not a catalytically competent substrate; thus, the structure obtained may not accurately reflect the O_2_-activating enzyme–substrate complex.

PCO and ADO variants at the equivalent position as the mouse Asp192 residue show abolished (or minimal) activity, indicating that this residue is catalytically essential (37, 38, 39). Based on this observation, it has been suggested that Asp192 may play a role in stabilizing or orientating reactive oxygen intermediates produced during catalytic turnover. The acidic Asp192 is expected to be deprotonated at physiological pH, and thus it cannot donate a hydrogen bond to transient iron-oxo intermediates (76) as proposed for CDO Tyr157 and MDO Tyr159 of the conserved SHY-motif (30). Numerous examples of conserved carboxylate residues (Asp and Glu) have been reported in mononuclear heme and non-heme iron oxygenases. However, to our knowledge, no other mononuclear non-heme iron enzyme features a carboxylate residue positioned as close to the catalytic Fe-site as ADO Asp192 while remaining non-coordinating.

For instance, within the active site of ethylene forming enzyme (PDB SV2Y) Glu84 forms strong hydrogen bonds with L-Arginine and is positioned 7.8 A from the Fe-site. Similarly, heme oxidase (PDB 1N3U) utilizes a conserved Asp140 residue in the active site pocket to orient solvent water and produce a stabilizing hydrogen bonding network in the active site. However, the position of Asp140 in human heme oxidase is 8.0 Å distant from the heme iron. In both examples, the transient oxygen intermediates produced at the Fe-site would be minimally influenced by electrostatic repulsion from the nearest carboxylate residue. By contrast, the anionic Asp192 is 2.7 to 2.9 Å distant from the Fe-site and thus would experience nearly 10-times the repulsive Coulombic force relative to heme oxidase. As demonstrated in Figure 7, the Mulliken charge of the atom coordinated trans to His100 plays a significant role in the stability of ligand binding at this position. Iron-oxo intermediates formed during reductive activation of dioxygen are expected to accumulate considerable anionic charge. Indeed, computational modes generated of an iron(III)-superoxide in substrate binding conformation [**4**] are thermodynamically unstable. It is therefore difficult to imagine how this position could represent a viable O_2_-binding site. Instead, we suggest that the anionic Asp192 helps attract and position the N_t_-alpha amine *trans* to His100. A similar argument was proposed by Fernández *et al*. on the basis of MD simulations (35). Further, as only the neutral substrate-amine can coordinate to the ferrous iron site, we speculate that Asp192 functions as a proton acceptor for N_t_-amine. This proton transfer also removes a negative charge from the active site, thereby favoring the subsequent reaction with molecular oxygen. Based on the models presented here, we propose that Asp192 plays an essential catalytic role in the assembly of a correct O_2_-activating enzyme-substrate complex rather than stabilizing transient iron-oxo intermediates.

### Mechanistic significance of NO binding studies

Thiol dioxygenases (TDOs) catalyze multi-substrate reactions involving both an organic substrate and molecular oxygen. Assuming nitric oxide (NO) and dioxygen exhibit analogous binding behavior, the order of substrate association can provide insight into the enzyme’s kinetic mechanism. In this context, ADO displays a marked difference in substrate-binding behavior compared to smTDOs (CDO and MDO). For these enzymes, the iron site is completely unreactive to NO in the absence of substrate. The binding of NO only occurs following anaerobic pre-incubation of the enzyme with its respective organic substrates (CYS or 3MPA) (24, 28, 42). This substrate-gated NO-reactivity has historically been used to demonstrate an obligate ordered kinetic mechanism with respect to dioxygen binding (24, 28, 29, 30, 31, 42, 77, 78, 79, 80).

In contrast, ADO readily reacts with NO in the absence of substrate to form an intermediate-spin (*S* = 3/2) iron–nitrosyl species (Fig. S6). Thus, NO binding at the ADO Fe-site is not dependent on prior substrate coordination. Subsequent addition of CA to the NO-bound ADO yields only trace quantities of the anticipated (CA/NO)-ADO complex; instead, treatment of NO–ADO with CA results in reduction of the iron–nitrosyl center. This behavior is more consistent with a preferred-order random kinetic mechanism. Whether this apparent difference in the kinetic mechanism of ADO reflects a substantive alteration in the underlying thiol dioxygenase chemical mechanism remains to be determined.

The systematic increase in ADO *k*_cat_ with increasing glycerol concentration (Fig. 2*A*) suggests that glycerol either enhances the rate of chemical steps in the reaction or increases the fraction of active enzyme present in solution. It is unlikely that increased viscosity from glycerol enhances the rate of either chemical or non-chemical steps, as both sucrose and PEG-200 vastly attenuate enzymatic activity. When considering the corresponding increase in bidentate (CA/NO)-ADO ternary complex observed upon treatment with glycerol (Fig. 5*B*), the most reasonable explanation for these observations is that glycerol activates ADO by increasing the fraction of active enzyme present. Furthermore, EPR spectroscopy detects iron–nitrosyl signals exclusively from the (CA/NO)-ADO ternary complex with bidentate CA coordination. This suggests that any enzyme present with monodentate CA binding is unreactive to nitric oxide, and by extension, dioxygen.

**Figure 9:**
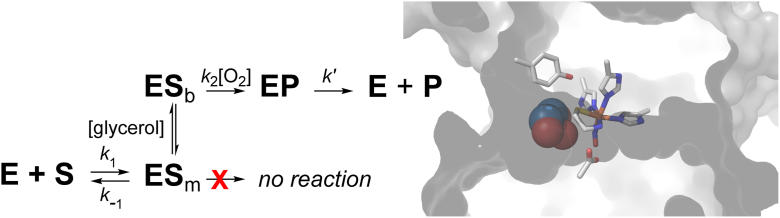
**Proposed kinetic model for glycerol-dependent activation of ADO.** (*left*) Proposed impact of glycerol on substrate-binding denticity. (*right*) ADO (PDB 7REI) active site substrate binding channel shown with glycerol (GOL308). Default van der Waal radii (sphere scale, 1.0) were used in Pymol ver. 3.1.6.1 for glycerol to illustrate scale of GOL308 relative to active site channel.

Based on these observations, we propose that the ADO enzyme-substrate complex exists in equilibrium between monodentate (**ES**_m_) and bidentate (**ES**_b_) CA coordination at the Fe-site (Fig. 9, *left*).

In this model, **ES**_m_ is catalytically inert and the observed activity is attributed solely to the amount of **ES**_b_ present in reactions. Molecular crowding from glycerol decreases the conformational space available for substrate binding at the Fe-site (Fig. 9, *right*), resulting in a shift in the equilibrium which favors bidentate CA-coordination. Consequently, the fraction of active enzyme (**ES**_b_) increases with added glycerol. In reactions with RGS N_t_-CYS substrates, the additional steric bulk of the trailing polypeptide segment likely provides an intrinsic crowding effect, resulting in a higher fraction of **ES**_b_ even in the absence of glycerol; as borne out empirically in EPR experiments.

The suppression of *k*_cat_ and *k*_cat_/K_M_ by added viscogens (sucrose and PEG-200) is consistent with rate-limiting product release. However, the absence of this behavior in reactions with glycerol is counterintuitive as it implies that diffusion limits are somehow overcome. This apparent paradox can be explained by an equilibrium shift that increases the concentration of active enzyme in proportion to the amount of glycerol present. Thus, diffusional limits are not overcome; rather, they are masked. Viscogens such as sucrose and PEG-200 do not alter the relative distribution of **ES**_m_ and **ES**_b_. Therefore, the effective concentration of active enzyme remains constant and kinetic solvent viscosity effects are observed.

### Summary

This work demonstrates that the catalytically active ES-complex of ADO exhibits bidentate substrate coordination through thiolate and amine functional groups, analogous to that observed for CDO. The results further show that glycerol (a common cryoprotectant and glassing agent) can significantly alter the *in vitro* activity of ADO by increasing the fraction of catalytically active enzyme. More broadly, these findings provide a cautionary example of how stabilizing agents, cryoprotectants, and glassing agents can influence enzymatic behavior when used without careful consideration of their mechanistic effects. Since the initial *in vitro* characterization of recombinantly expressed ADO in 2007, the nature of the ADO enzyme–substrate complex has remained a matter of considerable debate despite extensive spectroscopic, crystallographic, and mechanistic studies. Based on the work presented here, we argue that the longstanding ambiguity surrounding substrate binding at the ADO Fe site arises from three compounding factors. First, glycerol engages in direct, non-innocent interactions with the ADO enzyme–substrate complex; because differing amounts of glycerol have been used across research groups, the interpretation of results has been inconsistent. Second, prior studies made an unjustified assumption of equivalence between substrate binding to the oxidized, catalytically inactive Fe(III)-ADO and binding to the catalytically competent Fe(II)-ADO, an assumption we demonstrate here to be invalid. Third, ambiguity has been further compounded by variation in the choice of substrates employed across experimental studies (CA, RGS5, or CP6-LK8-Ser).

## Experimental procedures

### Protein expression & purification

The ADO expression vector was a generous gift from Prof. Thomas Brunold and Prof. Brian G. Fox (University of Wisconsin-Madison) (35, 40, 81). This vector codes for an N-terminal (His)_6_-motif followed by the small ubiquitin-related modifier (SUMO) fusion protein with *Mus musculus* cysteamine dioxygenase (ADO). A TEV protease site follows the His_6_-SUMO protein to allow for isolation of free ADO. The ADO expression vector was transformed into Rosetta2(DE3) competent *Escherichia coli* cells (Novagene) by heat shock. The cells were then grown overnight at 37 °C on an LB agar plate with both 50 mg/ml Kanamycin and 35 mg/ml chloramphenicol. The following day, a single colony was selected for growth in liquid LB (Kan/Cam) media for training on antibiotics prior to inoculation of 10-L BF-110 fermentor (New Brunswick Scientific) at 37 °C. Induction was initiated by adding 1.4 g isopropyl β-*D*-1-thiogalactopyranoside (IPTG), 78 mg ferrous ammonium sulfate, and 20 g casamino acids at an OD600 value of ∼4. During induction, the temperature of the bioreactor was lowered from 37 °C to 25 °C and agitation was set to maintain an oxygen concentration of 20% relative to air-saturated media. Cells were harvested and pelleted by centrifugation (Beckman-Coulter Avanti J-E, JA 10.5 rotor) once the growth reached an OD_600_ ∼ 2.0. The resulting cell paste was stored at −80 °C.

In a typical purification, 30*g* of frozen SUMO-ADO cell paste was added to the extraction buffer [20 mM 4-(2-hydroxyethyl)-1-piperazineethanesulfonic acid (HEPES), 50 mM NaCl, pH 8.0]. Lysozyme, ribonuclease, and deoxyribonuclease were added to the slurry for a final concentration of 10 μg/ml each and stirred slowly on ice prior to pulse sonication (Bronson Digital 250/450). The insoluble debris was removed from the cell free extract by centrifugation at 48,000*g* for 1 h at 4 °C. The ADO-SUMO fusion cell-free extract was purified using immobilized metal affinity chromatography (IMAC) with a gradient elution from 10 mM to 500 mM imidazole in a 20 mM HEPES, 350 mM NaCl, pH 8 buffer.

Fractions were collected using a Gilson FC 204 fraction collector, and the presence of the SUMO-ADO fusion protein was verified by 15% SDS-PAGE. Broad range protein molecular weight markers utilized in SDS PAGE experiments were purchased from Promega (Madison, WI) Cat. No. V8491. The pooled fractions were concentrated to approximately 5 to 10 ml using an Amicon stir cell equipped with a YM-10 ultrafiltration membrane.

To remove excess imidazole from the ADO-SUMO, a Sephadex G25 size exclusion column was used. ADO-SUMO was incubated with SUMO protease for 4 h at 4 °C to cleave the SUMO-tag from ADO. For ferric studies of ADO, a buffer exchange to 100 mM phosphate buffer, pH 7.4, was done using the same size exclusion column as mentioned earlier. The ADO in phosphate buffer was incubated with 5x excess sodium hexachloroiridate for 1 h at 4 °C before exchanging the buffer using a Sephadex G25 size exclusion column back to 20 mM HEPES, 50 mM NaCl, pH 8. Confirmation of ferric iron content was performed by the 2,4,6-tripyridyl-s-triazine (TPTZ) colorimetric assay described previously (24).

### Oxygen consumption

The rate of dioxygen consumption was determined polarographically using a standard Clark electrode (Hansatech Instruments, Norfolk, England) in a jacketed 2.5 ml cell. The electrode was bathed in a saturated solution of KCl and separated from the buffer using a gas-permeable membrane. Before use the electrode was calibrated as recommended by the product manufacturer. Reaction temperatures were maintained at 25 °C *via* a circulating water bath (Grant Instruments). Substrate was allowed to incubate in the electrode chamber for 30 s before reaction initiation by addition of ADO (5 μM). The reaction rate was observed for 60 s after the addition of ADO.

### Capillary electrophoresis-mass spectrometry (CE-MS) sample preparation

Two reaction mixtures were prepared, each containing 5 μM Fe(II)-ADO, 20 mM cysteamine hydrochloride, 20 mM HEPES, and 50 mM NaCl at pH 8.0. One solution contained 0% glycerol, while the other contained 55% glycerol. The reactions were incubated at room temperature, and aliquots were collected at 0, 5, 10, 30, and 60 min. At each time point, 200 μl of the reaction mixture was transferred to a 1.5 ml microcentrifuge tube and immediately heat-quenched at 100 °C for 10 min. The denatured samples were then centrifuged at 14,000 rpm for 10 min, and supernatants were collected and stored at −80 °C prior to analysis.

### CE-MS analysis

A 10 μl sample solution was diluted 10-fold with 90 μl of 5% acetic acid in HPLC water. Then, 20 nl of the diluted sample was injected using an ECE-001 CE autosampler from CMP Scientific (Brooklyn, NY) and analyzed by spray-capillary CE-MS, as described previously (82, 83, 84). All MS data were collected using Orbitrap mass spectrometers from Thermo Fisher Scientific (*e.g.*, the Exploris 240 MS or the Orbitrap Ascend MS) with a Nanospray Flex ion source. The reaction mixtures were analyzed using the Exploris 240 MS, while CE-MS method development and calibration curves were generated with the Orbitrap Ascend MS (details in Fig. S1). The MS inlet capillary temperature was set to 275 °C, and the electrospray ionization (ESI) voltage was adjusted to +1.5 kV for the Exploris 240 and + 1.7 kV for the Orbitrap Ascend. Mass spectra were obtained in small molecule mode for both mass spectrometers, with a default charge state of 1, a resolution setting of 120,000, and one microscan over a mass-to-charge (m/z) range from 50 to 200. The maximum injection time was set to automatic, and the automatic gain control (AGC) target value was set to standard. For the quantitative analysis of hypotaurine, extracted-ion electropherograms (EIEs) were generated for the m/z 110.0271 peak. The resulting peak was smoothed with a Gaussian function, and the integrated peak area was used for quantification.

### Solvent viscosity kinetic effects

For solvent viscosity studies, the steady-state kinetic parameters (*k*_cat_ and *k*_cat_/*K*_M_) were determined for ADO catalysis using the oxygen electrode at pH 8.0 (25 °C) described above. Experiments were performed using glycerol, sucrose, and PEG-200 to modify the buffer viscosity within reaction mixtures. The viscosity (η) of buffers was obtained using a Cannon-Fenske viscometer at 25 °C. The values obtained represent the average of triplicate measurements. Unless otherwise stated, the (%) composition of glycerol is reported in terms of a volumetric ratio (v/v).

### Data analysis

Steady-state kinetic parameters were determined by fitting data to the Michaelis-Menten equation using the program SigmaPlot ver. 15.0 (Systat Software Inc., Chicago, IL). From this analysis, both kinetic parameters (*k*_cat_ and K_M_) and error associated with each value were obtained by non-linear regression. The effect of solvent viscosity on the steady-state parameters was fit to Equation 1 (23, 85). In this expression, *v*_0_ represents each kinetic parameter (*k*_cat_ or *k*_cat_/K_M_) determined in the absence of viscogen whereas *v*_η_ is the value obtained at each specific relative viscosity measured. All viscosities measured are normalized to reaction buffer in the absence of viscogen (η_rel_). The slope of the line (*m*) represents the extent of diffusion limitation.

(Equation 1) $$\frac{v_{0}}{v_{\eta }}=1+m\cdot \eta_{rel\;}$$

### CD spectroscopy

Circular dichroism measurements were performed on a JASCO J-1500 CD spectrometer with a xenon arc light source. All experiments were performed using a quartz cuvette with a path length of 0.1 cm. For CD measurements, ADO was diluted to 8 μM (∼0.1 mg/ml) in 5 mM sodium phosphate buffer (pH 7.4) at varying glycerol concentrations. The spectrometer lamp was purged with ultra-pure nitrogen gas throughout the experiments. For each glycerol concentration, blank spectra of the buffer were obtained for subtraction. Spectra were collected within the UV region (190 nm to 300 nm) with five signal-averaged scans taken at 30 s each. Analysis of the CD spectra was done using the BeStSel CD analysis web tool (86, 87).

### Sample preparation for EPR & Mössbauer spectroscopy

CW EPR samples of mouse ADO were prepared in a glove box (Coy Laboratory Products Inc., Grass City, MI) with the O_2_ concentration maintained below 1 ppm. Solutions were degassed on a Schlenk line prior to transferring into the anaerobic chamber. Analytical grade argon was passed through a copper catalyst (Kontes) to remove trace dioxygen impurities and then sparged through distilled water to hydrate the gas. Stock solutions of cysteamine (CA) substrate were prepared in anaerobic 20 mM HEPES, 50 mM NaCl, pH 8.0. Equivalent EPR samples were prepared in which CA was substituted with a synthetic N_t_-CYS RGS5 polypeptide (CKGLAALPHSCLER), purchased from GenScript. Stock NO solutions were prepared by dissolving 5 mg of PROLI-NONOate (Cayman Chemical, 178,948–42–0) in a 200 μl solution of anaerobic 10 mM sodium hydroxide. Aliquots of degassed stock PROLI-NONOate (20–50 μl) were diluted 1:1 with deoxygenated buffer and allowed to react for 5 min. At pH 7.4, 2 mol of NO are released with each mol of NONOate with a half-life of 6 s at ambient temperature (88, 89). Samples of (*substrate*/NO)-bound enzyme were prepared anaerobically by the addition of 5 mol equivalents of 22 mM stock NONOate solution to enzyme pre-incubated with 10 mol equivalents of substrate. Samples were allowed to equilibrate on ice for 10 min prior to transferring the sample into an EPR tube using a 250 ml Hamilton gas-tight syringe equipped with a 6-inch needle. Finally, EPR tubes were frozen slowly in liquid N_2_ under anaerobic conditions prior to analysis. Quartz Supracil EPR sample tubes were made locally at the University of Alabama glassblowing facility operated by Rick Smith. The external diameter of EPR tubes used in CW and pulsed experiments was 4- and 3-mm, respectively.

Mössbauer samples were prepared under identical anaerobic conditions and reduced as described for EPR samples. All samples were prepared from a stock solution of ^57^Fe-enriched enzyme in which the Fe-content was verified by the colorimetric assay described above (*Iron Analysis*). For samples of the enzyme-substrate complex, 250 to 300 μl aliquots of the 1 to 3 mM enzyme were mixed with an anaerobic solution containing 10 to 15 mM CA titrated to the desired pH at 25 °C. Each sample was then frozen slowly (over 1–2 min) by immersion in liquid N_2_ within the anaerobic chamber.

Mössbauer spectra were collected at Carnegie Mellon University, Department of Chemistry. Data was recorded with a spectrometer operating in a constant acceleration mode in a transmission geometry using a Janis Research Inc. cryostat that allows for variation in temperature from 4 to 300 K. Isomer shifts are reported relative to Fe metal at 298 K. Spectral fitting was performed using SpinCount.

### Electron Paramagnetic Resonance (EPR) Spectroscopy

Continuous Wave (CW) EPR experiments were performed at the UA EPR facility using a Bruker ELEXSYS E540 X-band spectrometer (Bruker-Biospin Billerica, MA). Cryogenic measurements were made using a ColdEdge Stinger closed-loop liquid helium cryosystem inserted into an Oxford ESR900 cryostat. A LakeShore 336 temperature controller was used to regulate sample temperature. EPR simulations were calculated using SpinCount developed by Professor Michael Hendrich at Carnegie Mellon University by utilizing the general spin Hamiltonian as shown in Equation 2 (90, 91, 92).

(Equation 2) $$\hat{H}=D\left({\hat{S}}_{Z}^{2}-\frac{{\hat{S}}^{2}}{3}\right)+E\left({\hat{S}}_{X}^{2}-{\hat{S}}_{Y}^{2}\right)+\beta_{e}\cdot S\cdot \tilde{g}\cdot B+S\cdot \tilde{A}\cdot I$$

In this expression, *β*_*e*_ is the electron Bohr magneton, $\tilde{g}$ and $\tilde{A}$ are the *g*- and *A*-tensor, respectively for electronic (***S***) and nuclear spin (***I***), and the axial and rhombic zero-field splitting (*zfs*) parameters are represented by ***D*** and ***E****,* respectively. For all CW EPR simulations shown, hyperfine coupling is treated by second-order perturbation theory (93, 94).

For the low-spin ferric and iron-nitrosyl {FeNO} (7) (*S* = 1/2) signals, this expression simplifies to Equation 3.

(Equation 3) $$\hat{H}=\beta_{e}S_{c}\cdot \tilde{g}\cdot B+S\cdot \tilde{A}\cdot I$$

This program computes the powder pattern for a uniform spherical distribution of the magnetic field vector ***B***, and the transition intensities are calculated using ‘*Fermi’s golden rule*’ (95). All simulations were generated with consideration of all intensity factors, both theoretical and experimental, to allow for determination of species concentration. The only unknown factor relating spin concentration to signal intensity was an instrumental factor that is specific to the microwave detection system. However, this was determined by the spin standard, 1 mM Cu(EDTA), prepared from a copper atomic absorption standard solution purchased from Sigma-Aldrich.

### Pulsed EPR

Three pulse electron spin echo envelope modulation (3p ESEEM) spectra were measured using an ELEXSYS X-band E680 EPR spectrometer (Bruker-Biospin) equipped with a Bruker Flexline ER 4118 CF cryostat. 3p ESEEM measurements were made at 4 K at *g* = 2.08 (335 mT). Cryogenic measurements were made using the same ColdEdge closed-loop LHe cryosystem described for CW EPR measurements. Data were collected using a three-pulse stimulated echo sequence, π/2−τ−π/2 − T−π/2−τ+T−echo, where π/2 represents a 16 ns microwave pulse, and T and τ represent delays between the pulses. Delay times (τ) were selected based on the maximum (234 nsec) and minimum intensity of ^1^H-modulation (208 nsec) observed in the 2p ESEEM experiment, and the pulse sequence was repeated at a rate of 1.5 kHz.

ESEEM data were processed using standard functions in xEPR (Bruker). Briefly, the complex raw data were phased to minimize the imaginary component, then converted to a natural log scale before subtracting background decay using a second-order polynomial. A cosbell apodization window function was applied to the time domain data to minimize noise at longer values of T. The data were then zero-filled to 2048 points before calculating the Fourier transform. Difference ESEEM spectra were obtained by subtraction of processed time domain data prior to Fourier transform.

### Computational modeling

Density functional theory (DFT) calculations were done using ORCA version 6.0 (96). Starting coordinates for geometry optimizations were obtained from the crystal structures for *M. musculus* and human ADO (PDB code 7LVZ and 7REI). In addition to the iron and directly coordinated histidine residues (His100, His102, and His179), outer-sphere residues Asp192, Tyr198, and Cys206 were included to provide stabilizing interactions with cysteamine (CA) and nitric oxide (NO). ChemCraft (ver 1.8) was used to add co-substrates to crystallographic coordinates and model the six possible binding conformers. The models were further edited by capping alpha carbons for each residue with methyl groups. Iron-coordinated histidine residues were capped at the β-carbon and protonated at the δ-position. Geometry optimization of the (*S* = 1/2) iron-nitrosyl complexes used the B3LYP functional with the Ahlrichs def2-tzvp basis set on iron and directly coordinated atoms and the def2-svp basis set on all other atoms (97, 98, 99). EPR parameters were calculated on the most stable conformation using the EPR-II functional. All calculations utilized Grimme’s D3 dispersion correction, a CPCM solvent model with ε = 4, to emulate a protein environment, and the resolution of identity and chain of sphere (RIJCOSX) approximation for B3LYP with def2/J auxiliary basis sets (100, 101, 102, 103).

## Data availability

Atomic coordinates for lowest energy (CA/NO)-ADO computational model (conformation [**3**]) are provided in supporting information, Table S7. All other molecular coordinates, computational results, and raw spectroscopic data used in this study will be made available upon request (Contact: Brad Pierce, bspierce1@ua.edu).

## Supporting information

This article contains supporting information (21, 28, 41, 42, 51, 55, 56, 57, 58, 72, 84).

## Conflict of interest

The authors declare that they do not have any conflicts of interest with the content of this article.
