# Supplementary material for: EPR spectroscopy reveals glycerol-dependent activation of cysteamine dioxygenase (ADO) enables bidentate substrate coordination
Source: J Biol Chem. 2026 Apr 14;302(6):111438. doi: 10.1016/j.jbc.2026.111438 (PMC13194613; doi:10.1016/j.jbc.2026.111438)
Supplement: Supplementary material [file mmc1.pdf]

Supporting Information

EPR spectroscopy reveals glycerol-dependent activation of cysteamine dioxygenase (ADO) enables bidentate substrate coordination

*Joshua R. Helms[1]; Miriam Probst[1]; Jared Paris[2]; Patrycja Szamweber[1], Zhitao Zhao[1], Si Wu[1], Brad S. Pierce[1]**

1. Department of Chemistry & Biochemistry, University of Alabama, 250 Hackberry Lane, Tuscaloosa, Alabama 35487, United States

2. Department of Chemistry, Carnegie Mellon University, 4400 Fifth Avenue, Pittsburgh, PA 15213, United States

AUTHOR INFORMATION

Corresponding Author(s)
* To whom correspondence should be addressed:

Brad S. Pierce, Department of Chemistry & Biochemistry, University of Alabama, Tuscaloosa, AL 35487, USA, Telephone, (205)348-8445, Email: bspierce1@ua.edu

Table of Contents for Supplementary Information

Contents	Page
**FIGURES**	
**Figure S1**. **A**. Mass spectra of experimental and theoretical isotopic envelope of hypotaurine. **B**. extracted ion electropherogram (EIE) of hypotaurine from CE-MS analysis. **C**. calibration curve of relative intensity of hypotaurine (n = 3) without glycerol.	2
**Figure S2**. **A**. Solvent viscosity kinetic effects on the maximal rate of ADO catalyzed **HT** formation in the presence of sucrose and PEG-200. **B**. comparison of kinetic parameters obtained in the presence of sucrose, PEG-200, BSA, glycerol, and glyceraldehyde.	3
**Figure S3**. UV Circular dichroism (CD) spectra of ADO with 0%, 5% , 25%, and 55% (v/v) glycerol content.	3
**Figure S4**. Mössbauer doublets observed in mouse Fe(II)-ADO in the absence and presence of excess CA.	4
**Figure S5**. Temperature normalized signal area (S×T) measured for samples CA-bound Fe(III)-ADO and CYS-bound Fe(III)-ADO.	5
**Figure S6**. EPR spectra and temperature normalized signal area (S×T) measured for intermediate spin (S = 3/2) iron-nitrosyl ADO form in the absence of substrate.	7
**Figure S7**. Microwave power saturation data for observed iron-nitrosyl species.	8
**Figure S8**. **A**. Representative CW EPR spectra for Fe(II)-ADO treated with NO in the absence of CA. **B**. Representative spectra illustrating the change in relative intensity for $\{FeNO\}^7$ and DNIC upon titration with CA. **C**. Quantitation of $\{FeNO\}^7$ (S = 3/2) and DNIC (S = 1/2) signals with increasing CA-concentration.	9
**Figure S9**. Alignment of ADO crystal structures (PDB code: 7REI, 8UAN, and 8U9J)	10
**TABLES**	
Table S1. *Analysis of ADO secondary structure (%) with increasing glycerol content.*	11
**Table S2**. *Comparison of Fe(II)-ADO and CA-Fe(II)-ADO Mössbauer parameters to other thiol dioxygenase enzymes (CDO and MDO).*	12
Table S3. *EPR simulation parameters used for Fe(III)-ADO species.*	12
**Table S4**. Microwave power at half-saturation for EPR observed nitrosyl species.	13
Table S5. *Comparison of selected bond distances and angles for ADO x-ray crystal structures (PDB code 7LVZ and 7REI).*	13
Table S6. *Comparison of experimental and calculated EPR parameters for (CA/NO)-bound ADO.*	13
**Table S7**. Coordinates for (CA/NO)-ADO in substrate-binding conformation [**3**]	15

FIGURES

Figure S1

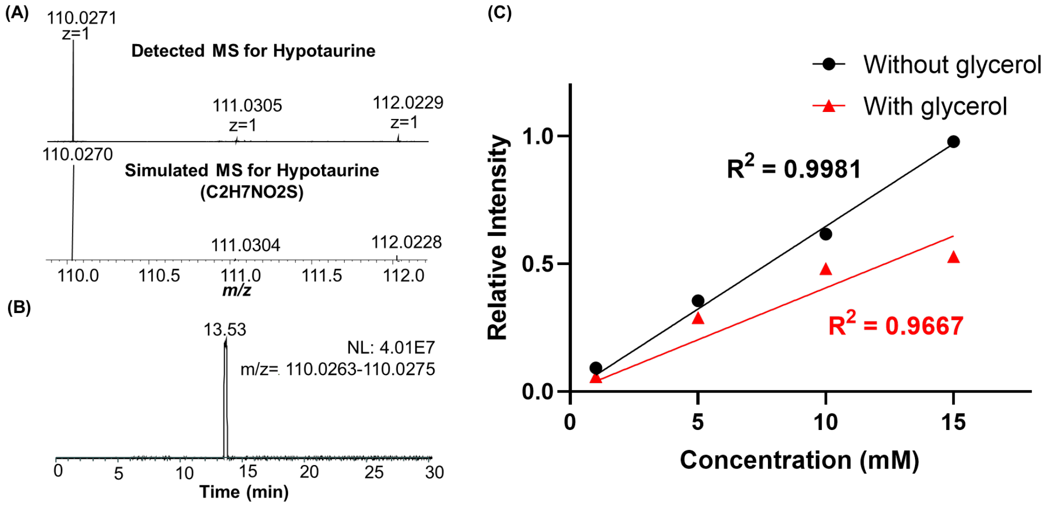

Figure S1. (A) Mass spectra of experimental and theoretical isotopic envelope of hypotaurine. (B) Extracted ion electropherogram (EIE) of hypotaurine from CE-MS analysis. (C) Calibration curve of relative intensity of hypotaurine (n = 3) with and without glycerol.

Standard hypotaurine solutions at varying concentrations (1, 5, 10, and 15 mM) were prepared with and without glycerol. A 10 μL sample solution was diluted 10-fold with 90 μL of 5% acetic acid in HPLC water and 20 nL of the diluted sample was injected and evaluated using our spray-capillary CE-MS platform (**Figure S1**). Using high-resolution Orbitrap MS, we successfully detected hypotaurine (m/z = 110.0271) with high mass accuracy. As illustrated in **Figure S1A**, the experimental high-resolution mass spectrum (top) displays an isotopic envelope that matches the theoretically predicted distribution calculated from the empirical formula of hypotaurine ($C_2H_7NO_2S$, $[M+H]^+$, bottom), thereby providing confident molecular identification. Furthermore, the EIE shown in Figure S1B exhibits a distinct, well-defined peak at 13.53 minutes, suggesting that our spray-capillary CE-MS platform can efficiently separate hypotaurine from the sample matrix for quantitative analysis.

To further evaluate the quantitative performance of the CE-MS platform for hypotaurine, calibration curves were generated in the presence and absence of glycerol. Calibration curves were constructed by plotting hypotaurine concentration against the normalized peak area. Both calibration curves showed good linear correlation (R^2 = 0.9981 for standards in the absence of glycerol and R^2 = 0.9667 for standards in the presence of glycerol). As shown in **Figure S1C**, samples prepared without glycerol consistently produced higher signal intensities compared with those containing glycerol. This may be due to slightly different sampling rates caused by viscosity variations during hydrodynamic sample injection in CE-MS.[1-3] Therefore, in the analysis of the reaction mixture, we normalized peak intensities using the slope of calibration curves with glycerol compared to those without glycerol.

Figure S2

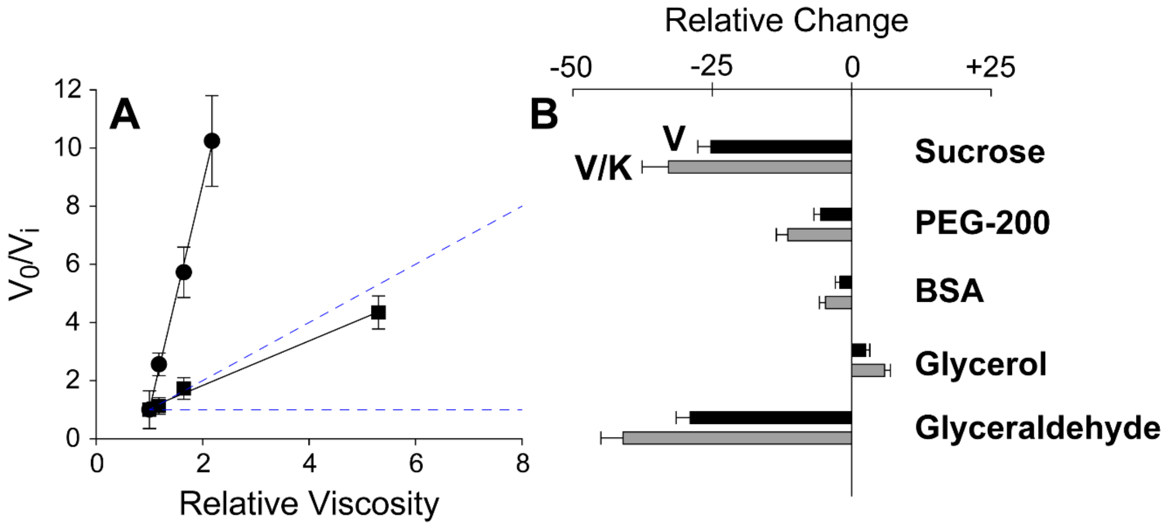

Figure S2. **A**. Solvent viscosity kinetic effects on the maximal rate (v_0/[E]) of ADO catalyzed **HT** formation in the presence of sucrose (●) and PEG-200 (■). The dashed blue lines indicate the theoretical limits for diffusion-limited product release. **B**. comparison of kinetic parameters obtained in the presence of sucrose, PEG-200, BSA, glycerol, and glyceraldehyde.

Figure S3

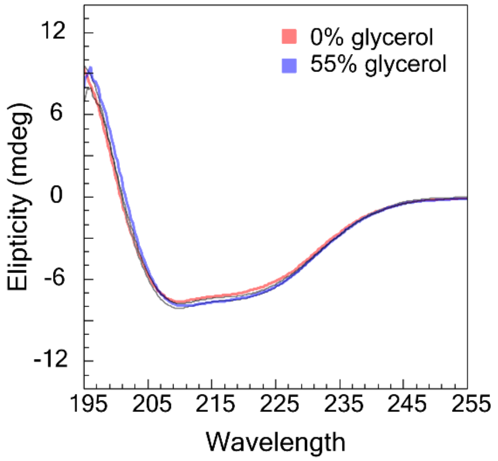

Figure S3. UV Circular dichroism (CD) spectra of 0.1 mg/mL (8 μM) ADO with 0% (*red*), 5% (*gray*), 25% (*gray*), and 55% (*blue*) glycerol content. Analysis of ADO secondary structure from CD spectra provided in **Table S1**.

Figure S4

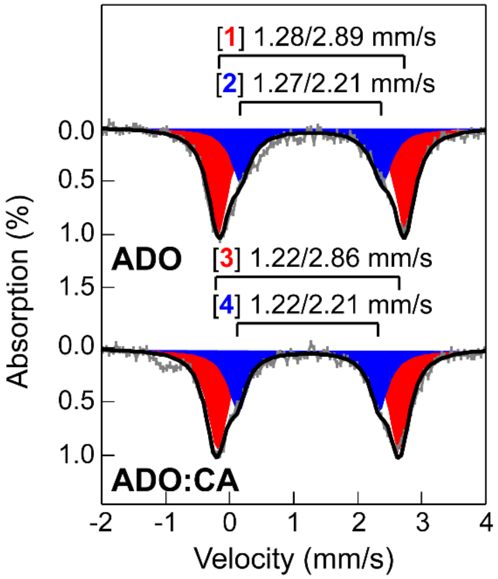

Figure S4. (**A**) Mössbauer doublets observed in mouse Fe(II)-ADO in the absence (*top*) and presence of excess CA (*bottom*). **Table S2** summarizes the data observed in the presence and absence of 55% glycerol.

Supporting EPR studies.

The energy separating the ground doublet from excited states for an S = 5/2 spin center was obtained experimentally by fitting the temperature-normalized signal area (S×T) for data collected across a broad temperature range (4 - 30 K) to a Boltzmann population distribution for a 3-level system (**Equation S1**).

$$\boldsymbol{S \times T \sim n_s} = \frac{g_i \cdot e^{-\Delta E_i/k_b T}}{\sum_j g_j \cdot e^{-\Delta E_j/k_b T}} = \frac{(2S_i+1)\cdot e^{-DS_{z,i}^2/k_b T}}{\sum_j (2S_j+1)\cdot e^{-DS_{z,j}^2/k_b T}} \qquad \textbf{Equation S1}$$

Figure S5

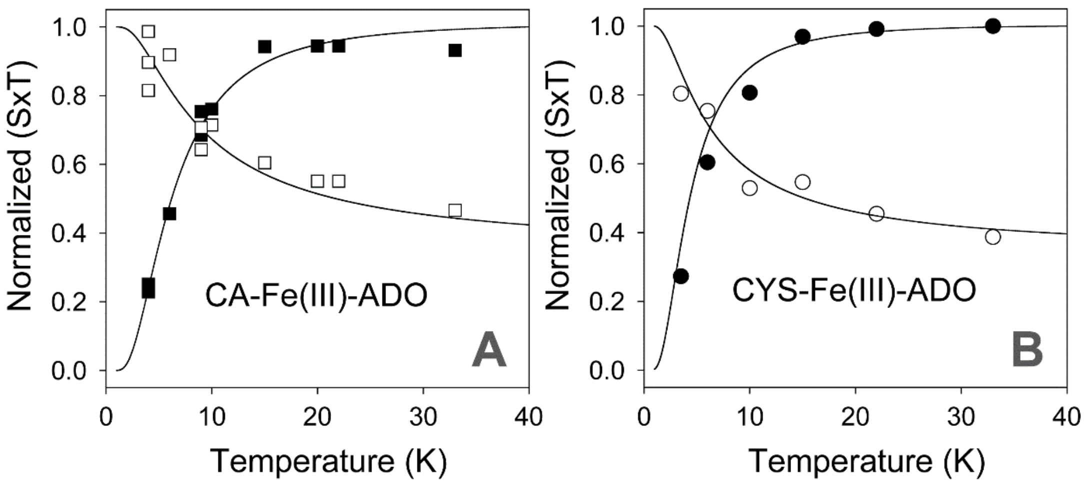

Figure S5. Temperature normalized signal area (S×T) measured for samples CA-bound Fe(III)-ADO (**A**, S = 5/2) and CYS-bound Fe(III)-ADO (**B**, S = 5/2). For high-spin (S = 5/2) sites, the filled marker represents the population curve for the middle doublet (levels, 3-4) and the empty marker represents the ground doublet, levels 1-2. Data fits (*overlaid solid and dashed lines*) to a Boltzmann population distribution for a 3-level system.

Titration of NO-bound ADO with excess CA. Experiments probing the order of substrate and NO addition reveal a marked difference between ADO and smTDOs (CDO and MDO) previously characterized by EPR.[4-6] Both CDO and MDO display a strict obligate-ordered mechanism in which the organic substrate binds prior to NO. In contrast, treatment of ADO with excess NO in the absence of substrate yields a nearly axial (E/D = 0.016) intermediate-spin (S = 3/2) iron–nitrosyl species, as observed by EPR (**Figure S6**).

Subsequent titration of this NO-bound ADO with CA leads to loss of the S = 3/2 signal and the concomitant appearance of a sharp S = 1/2 signal with observed g-values of 2.04, 2.02, and 2.00 (g_{av} = 2.03). This spectrum matches that of a well-characterized $Fe(NO)_2L_2$ species known as a dinitrosyl iron complex (DNIC). On the basis of its formation upon

addition of CA to NO-bound ADO, it was previously reported by Wang *et al*. that this DNIC arises from simultaneous binding of two molar equivalents of NO and monodentate CA at the 3-His iron site of ADO, thereby forming a six-coordinate DNIC.[7] As discussed below, several lines of evidence argue against this interpretation.

First, DNICs are well-known contaminants that readily form upon addition of NO to samples containing iron.[8, 9] Such complexes, including those formed with CYS and CA, have been extensively characterized by crystallographic, spectroscopic, and computational methods.[10-16] Importantly, DNICs that exhibit the characteristic EPR signal with g_{av} = 2.03 are exclusively tetrahedral, four-coordinate species.[15] Although a small number of six-coordinate DNICs have been reported, these display reduced *g*-anisotropy, with g_{av} values of 2.013.[14, 16] Moreover, it is notoriously difficult to differentiate DNICs arising from adventitious reactions in solution versus those produced by excess NO binding to an enzymatic metal center.[17]

Second, in our earlier characterization of the (CYS/NO)-CDO system, we demonstrated that an identical DNIC EPR signal with g_{av} = 2.03 could be generated by adding NO to aqueous solutions containing only CYS and Fe(II), in the absence of CDO.[5] This control established that the DNIC signal did not originate from the enzyme but instead from nonspecific reactions among NO, adventitious Fe(II), and free CYS.[18, 19] Notably, this critical control experiment was not included in the work reported previously.[7]

DNICs generated in aqueous solutions of Fe(II), CA, and NO exhibit *g*-values (**Figure 5**) and microwave power at half-saturation ($P_{1/2}$) (**Figure S7**) that are indistinguishable from those observed in samples of (CA/NO)-ADO. Indicating that this signal originates from the same chemical species. Because this DNIC signal is produced in the complete absence of ADO, it cannot be attributed to simultaneous binding of CA and NO at the ADO active site.

This conclusion is further supported by quantitative analysis of the EPR spectra obtained during titration with excess CA. As shown in **Figure S8**, addition of CA to NO-bound ADO results in progressive loss of the S = 3/2 iron–nitrosyl signal. For each titration point, the concentration of NO-bound ADO was determined by analytical EPR simulations that incorporate the experimentally determined (**Figure 6B**) axial zero-field splitting parameter (D = 7.6 ± 0.4 cm^{-1}). In parallel, formation of the S = 1/2 DNIC was monitored throughout the titration. As shown in **Figure S8C**, treatment with a 13-fold molar excess of CA relative to NO-bound ADO nearly abolishes the S = 3/2 signal. Under these same conditions, however, less than 10 percent of the total iron is accounted for as DNIC. Thus, the extent of iron–nitrosyl loss far exceeds the amount of DNIC formed.

This behavior is inconsistent with direct binding of CA to NO-bound ADO, as previously proposed.[7] Instead, the data indicate formation of a major EPR-silent product. Given that CA contains a redox-active thiol, the most straightforward explanation is reduction of the

NO-bound iron center in ADO by CA, which is a known reducing agent. Once reduced, excess CA can chelate ferrous iron from the active site of ADO resulting in adventitious iron as suggested by the increase in DNIC formation. Accordingly, all experiments performed confirm that the DNIC signals observed in samples of (CA/NO)-ADO are unrelated to substrate binding at the ADO active site and instead arise from nonspecific reactions among NO, adventitious Fe(II), and free CA ligand.

Figure S6

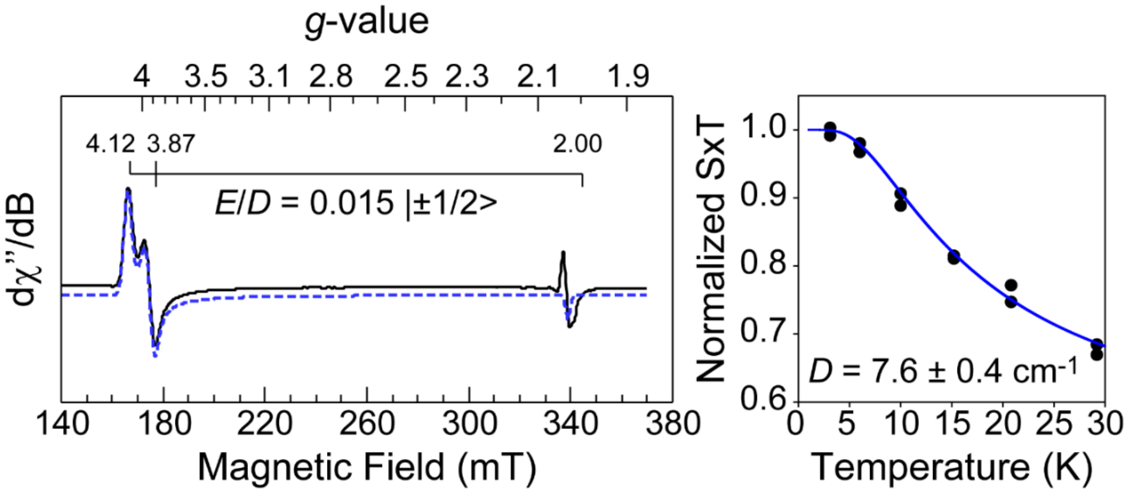

Figure S6. EPR spectra and temperature normalized signal area (S×T) measured for intermediate spin (S = 3/2) iron-nitrosyl ADO form in the absence of substrate. Data fit (*overlaid solid blue line)* to a Boltzmann population distribution for a 2-level system.

Half-power microwave saturation ($P_{1/2}$) values reported for selected samples were determined by collecting scans at increasing microwave power and fixed scan rate and field width. The signal area as a function of microwave power was fit using the SpinCount software package according to **equation S2**.

$$S/\sqrt{P} = \frac{A}{\left(1 + P/P_{1/2}\right)^{b/2}} \qquad \textbf{equation S2}$$

The software performs least-squares fitting of the normalized derivative signal intensity (S) as a function of microwave power (P). The A-term represents the normalized maximum signal amplitude. The variable b is a spectroscopic inhomogeneity factor which is characteristic of the spin packet of the observed resonance. The signal derived from frozen solutions and powders typically exhibit inhomogeneous line broadening behavior in which b = 1.

Figure S7

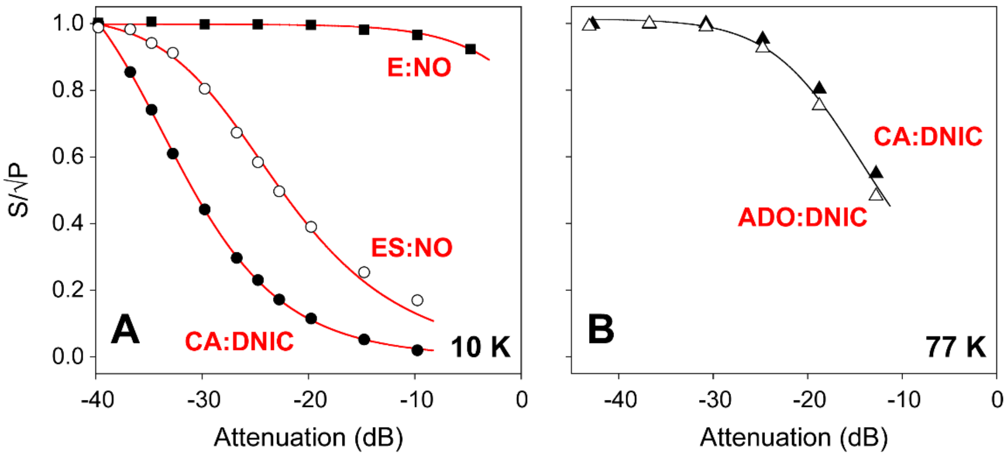

Figure S7. Microwave power saturation data for observed iron-nitrosyl species were fit to **Equation S2**. A summary of the microwave power at half-saturation is provided in **Table S4**.

Figure S8

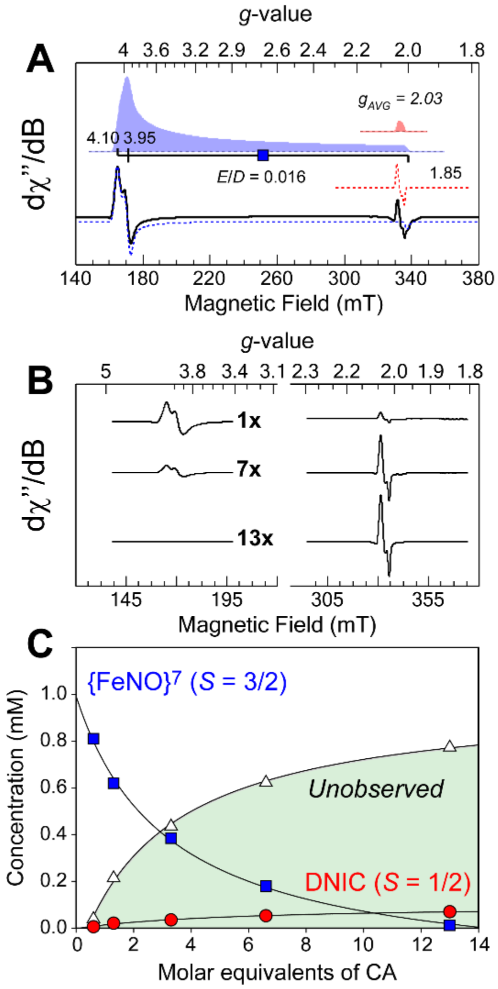

Figure S8. **A**. Representative 10 K CW EPR spectra for Fe(II)-ADO treated with NO in the absence of CA. The observed spectra show two signals. The first of which can be assigned to an intermediate spin (*S* = 3/2) $\{FeNO\}^7$ with observed *g*-values of 4.10, 3.95, and 2.00. The second signal (g_{AV} ~ 2.03) is equivalent to monomeric DNIC (*S* = 1/2) shown in **Figure 5**. For ease of comparison, the area for each species is highlighted in blue and red, respectively. **B**. Representative spectra illustrating the change in relative intensity for $\{FeNO\}^7$ and DNIC upon titration with CA. **C**. Quantitation of $\{FeNO\}^7$ (*S* = 3/2) and DNIC (*S* = 1/2) signals with increasing CA concentration. The filled area (*green*) illustrates the fraction of iron not observed as either $\{FeNO\}^7$ or DNIC. **Table S3** summarizes the EPR simulation parameters obtained and the experimentally determined iron-nitrosyl *D*-value (**Figure S6**).

Figure S9

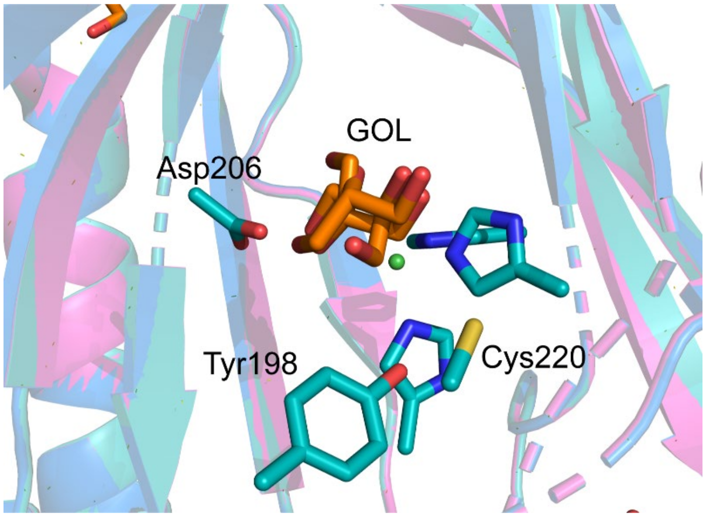

Figure S9. Alignment of ADO crystal structures (PDB code: 7REI, 8UAN, and 8U9J) highlighting the overlap of GOL308, GOL302, and GOL302 (*orange*). This is the same glycerol molecule superimposed with the substrate-bound computational model [**3**] shown in **Figure 8**.

TABLES

***Table S1**. Analysis of ADO secondary structure (%) with increasing glycerol content.*

Glycerol (%)	α-helix	Antiparallel	Parallel	Turn	Other	Total
0	59	0	3	8	30	100
5	64	0	0	8	29	100
25	62	0	2	9	28	100
55	68	0	2	8	22	100

CD spectra analyzed using the BESTSEL website (https://bestsel.elte.hu/results.php) within the range of 200 – 250 nm.

Table S2. *Comparison of Fe(II)-ADO and CA-Fe(II)-ADO Mössbauer parameters to other thiol dioxygenase enzymes (CDO and MDO).*

		fraction	(mm/s)		
**Enzyme**		(%)	δ	ΔE_Q	Γ
			**No glycerol**		
ADO					
	**1**	75	1.28	2.89	0.4
	**2**	25	1.28	2.21	0.4
ADO:CA					
	**3**	76	1.22	2.86	0.4
	**4**	24	1.22	2.21	0.4
			**55% glycerol**		
ADO					
	**1'**	83	1.27	2.91	0.4
	**2'**	17	1.27	2.21	0.4
ADO:CA					
	**3'**	85	1.21	2.86	0.4
	**4'**	15	1.21	2.21	0.4
			**No glycerol**		
CDO[20, 21]					
	**1**	100	1.22	2.85	0.43
CDO:CYS					
	**2**	40	1.03	2.86	0.43
	**3**	60	1.10	3.14	0.61
MDO[6, 22]					
	**1**	54	1.21	2.45	0.4
	**2**	46	1.24	2.90	0.4
MDO:3MPA					
	**1**	50	1.06	1.79	0.4
	**2**	50	1.07	2.24	0.4

Table S3. *EPR simulation parameters used for Fe(III)-ADO species.*

Sample	Spin	D (cm^{-1})[a]	E/D	g_x	g_y	g_z	σ_B (mT)
CA-Fe(III)-ADO	5/2	2.2 ± 0.3	0.23	2.00	1.98	2.00	0.7
CYS-Fe(III)-ADO	5/2	1.6 ± 0.3	0.25	1.98	2.02	2.03	0.7
CYS-Fe(III)-ADO	1/2	-	-	2.40	2.31	1.91	0.7
NO-ADO	3/2	7.6 ± 0.6	0.015	2	2	2	0.7

NOTES: [a]The magnitude of *D* was determined experimentally by measuring the temperature-dependent signal intensity and fitting to an appropriate Boltzmann distribution curve.

Table S4. Microwave power at half-saturation for EPR observed nitrosyl species. Values were obtained by fitting data collected at 10 and 77 K (**Figure S7**) to **Equation S2**.

Signal	Spin	$P_{1/2}$ (mW)	T (K)
E:NO	3/2	400	10
ES:NO	1/2	0.35	10
CA:DNIC	1/2	0.05	10
CA:DNIC	1/2	3.80	77
ADO:DNIC	1/2	3.80	77

***Table S5**. Comparison of selected bond distances and angles for ADO x-ray crystal structures (PDB code 7LVZ and 7REI). These represent the starting coordinates for each DFT model.*

Bond	DFT (7LVZ) r(Å)	DFT (7REI) r(Å)	Bond Angle	DFT (7LVZ) ∠(deg)	DFT (7REI) ∠(deg)
Fe-Nε(H100)	2.03	2.03	(H179)Nε-Fe-N(CA)	98.8	95.6
Fe-Nε(H102)	2.25	2.20	(H100)Nε-Fe-Nε(H102)	89.4	89.7
Fe-Nε(H179)	2.23	2.09	(H100)Nε-Fe-S(CA)	85.2	87.5
Fe-S (CA)	2.31	2.29	(H102)Nε-Fe-S(CA)	91.9	93.1
Fe-N (CA)	2.07	2.07	(CA)N-Fe-S(CA)	86.1	86.6
Fe-N (NO)	1.73	1.74	Fe-N-O(NO)	144.3	144.2
N-O (NO)	1.17	1.17	(H179)Nε-Fe-N(NO)	93.2	92.4
			(H100)Nε-Fe-Nε(H179)	89.6	90.1
			(H102)Nε-Fe-Nε(H179)	82.2	84.6

***Table S6**. Comparison of experimental and calculated EPR parameters for (**CA**/NO)-bound ADO.*

Structure	Initial Coordinates	Resolution (Å)	g_x	g_y	g_z	A_x (MHz)	A_y (MHz)	A_z (MHz)
DFT/BP86	7LVZ	1.89	2.038	2.009	1.982	35	65	28
DFT/BP86	7REI	1.78	2.034	2.006	1.974	34	70	29
Experimental								
(CA/NO)-ADO			2.086	2.018	1.982	30	74	34
(RGS5/NO)-ADO			2.081	2.019	1.981	37	68	30

Table S7. Atomic Coordinates for (CA/NO)-ADO in conformation [**3**]

C	33.285474000000	56.461130000000	94.768572000000
C	32.010896000000	56.075664000000	95.114574000000
N	33.628307000000	55.652110000000	93.701447000000
C	32.590639000000	54.828822000000	93.427004000000
N	31.599711000000	55.058189000000	94.271986000000
H	33.711399000000	58.121949000000	96.068414000000
H	34.739655000000	58.062433000000	94.607906000000
H	34.507312000000	55.678900000000	93.194135000000
H	31.363934000000	56.473464000000	95.887854000000
H	32.582663000000	54.100347000000	92.621707000000
C	28.116932000000	56.017686000000	99.511579000000
C	28.786713000000	55.355826000000	98.354979000000
C	28.491364000000	55.308231000000	97.010746000000
N	29.961229000000	54.636613000000	98.479360000000
C	30.328059000000	54.187416000000	97.255058000000
N	29.459077000000	54.582444000000	96.340299000000
H	27.905603000000	55.295987000000	100.328790000000
H	27.156213000000	56.467134000000	99.198088000000
H	30.465061000000	54.467852000000	99.344263000000
H	27.657316000000	55.768593000000	96.495992000000
H	31.218024000000	53.593512000000	97.076528000000
C	33.077968000000	50.050737000000	96.433373000000
C	31.933251000000	50.786106000000	95.793970000000
C	31.851904000000	52.042009000000	95.225261000000
N	30.642432000000	50.293069000000	95.718921000000
C	29.846988000000	51.228529000000	95.138045000000
N	30.548074000000	52.303636000000	94.830905000000
H	33.337717000000	49.104249000000	95.913927000000
H	32.847642000000	49.795450000000	97.489463000000
H	30.335032000000	49.379671000000	96.039451000000
H	32.644539000000	52.770726000000	95.093618000000
H	28.782434000000	51.089780000000	94.965643000000
C	23.908596000000	52.882777000000	90.580251000000
C	24.733987000000	52.861876000000	91.898549000000
O	24.147235000000	53.294070000000	92.921156000000
O	25.906283000000	52.397492000000	91.838683000000
H	24.560577000000	52.820116000000	89.683808000000
H	23.324354000000	53.822200000000	90.483417000000
C	25.912173000000	62.835708000000	95.971666000000
C	26.765243000000	61.589120000000	96.002336000000
C	27.381066000000	61.165390000000	97.191022000000
C	26.998867000000	60.822987000000	94.847606000000
C	28.202183000000	60.036544000000	97.230708000000
C	27.821010000000	59.694013000000	94.868178000000

C	28.436982000000	59.292356000000	96.066517000000
O	29.290395000000	58.240127000000	96.140183000000
H	26.346648000000	63.610763000000	95.306955000000
H	25.835939000000	63.263658000000	96.991099000000
H	27.219104000000	61.736689000000	98.110039000000
H	26.537896000000	61.122116000000	93.901239000000
H	28.682974000000	59.722082000000	98.160333000000
H	28.010993000000	59.133803000000	93.950792000000
H	29.237702000000	57.664154000000	95.322796000000
C	30.763245000000	61.836572000000	95.260695000000
S	31.540908000000	60.394045000000	94.434547000000
H	30.916401000000	61.785677000000	96.355705000000
H	29.680819000000	61.893602000000	95.036003000000
H	30.870082000000	59.427909000000	95.103202000000
Fe	29.741038000000	54.255906000000	94.131786000000
N	29.921683000000	53.843164000000	92.459559000000
H	23.212964000000	52.020287000000	90.595283000000
H	24.878528000000	62.645269000000	95.611129000000
H	35.015682000000	56.891187000000	95.936154000000
H	31.268019000000	62.739518000000	94.867567000000
H	33.974682000000	50.698273000000	96.427936000000
H	28.748352000000	56.825426000000	99.938324000000
S	29.056618000000	56.412126000000	93.648478000000
C	27.251326000000	56.178291000000	93.786825000000
H	26.744893000000	56.912330000000	93.142999000000
H	26.917422000000	56.375096000000	94.817203000000
H	27.078995000000	54.672868000000	92.266062000000
C	26.891291000000	54.782334000000	93.340148000000
H	25.830463000000	54.538767000000	93.503085000000
N	27.733515000000	53.746298000000	94.017439000000
H	27.405151000000	53.617139000000	94.971658000000
H	27.499929000000	52.880869000000	93.522074000000
O	29.430539000000	53.929083000000	91.403399000000

REFERENCES

(1) Huang, L.; Wang, Z.; Cupp-Sutton, K. A.; Smith, K.; Wu, S. Spray-Capillary: An Electrospray-Assisted Device for Quantitative Ultralow-Volume Sample Handling. *Analytical Chemistry* **2020**, *92* (1), 640-646.

(2) Chen, A.; Lynch, K. B.; Ren, J.; Jia, Z.; Yang, Y.; Lu, J. J.; Liu, S. Tunable electroosmosis-based femto-liter pipette: a promising tool toward living-cell surgery. *Analytical Chemistry* **2017**, *89* (20), 10806-10812.

(3) Breadmore, M. C. Electrokinetic and hydrodynamic injection: making the right choice for capillary electrophoresis. *Bioanalysis* **2009**, *1* (5), 889-894.

(4) Pierce, B. S.; Subedi, B. P.; Sardar, S.; Crowell, J. K. The "Gln-Type" Thiol Dioxygenase from Azotobacter vinelandii Is a 3-Mercaptopropionic Acid Dioxygenase. *Biochemistry* **2015**, *54* (51), 7477-7490.

(5) Pierce, B. S.; Gardner, J. D.; Bailey, L. J.; Brunold, T. C.; Fox, B. G. Characterization of the Nitrosyl Adduct of Substrate-Bound Mouse Cysteine Dioxygenase by Electron Paramagnetic Resonance: Electronic Structure of the Active Site and Mechanistic Implications. *Biochemistry* **2007**, *46* (29), 8569-8578.

(6) Sardar, S.; Weitz, A.; Hendrich, M. P.; Pierce, B. S. Outer-Sphere Tyrosine 159 within the 3-Mercaptopropionic Acid Dioxygenase S-H-Y Motif Gates Substrate-Coordination Denticity at the Non-Heme Iron Active Site. *Biochemistry* **2019**, *58* (51), 5135-5150.

(7) Wang, Y.; Davis, I.; Chan, Y.; Naik, S. G.; Griffith, W. P.; Liu, A. Characterization of the nonheme iron center of cysteamine dioxygenase and its interaction with substrates. *J Biol Chem.* **2020**, *295* (33), 11789-11802.

(8) Foster, M. W.; Cowan, J. A. Chemistry of Nitric Oxide with Protein-Bound Iron Sulfur Centers. Insights on Physiological Reactivity. *J Am Chem Soc.* **1999**, *121* (17), 4093-4100.

(9) Tinberg, C. E.; Tonzetich, Z. J.; Wang, H.; Do, L. H.; Yoda, Y.; Cramer, S. P.; Lippard, S. J. Characterization of Iron Dinitrosyl Species Formed in the Reaction of Nitric Oxide with a Biological Rieske Center. *J Am Chem Soc.* **2010**, *132* (51), 18168-18176.

(10) Ye, S.; Neese, F. The Unusual Electronic Structure of Dinitrosyl Iron Complexes. *J Am Chem Soc.* **2010**, *132* (11), 3646-3647.

(11) Sellmann, D.; Blum, N.; Heinemann, F. W.; Hess, B. A. Synthesis, Reactivity, and Structure of Strictly Homologous 18 and 19 Valence Electron Iron Nitrosyl Complexes. *Chem. Eur. J.* **2001**, *7* (9), 1874-1880.

(12) Enemark, J. H.; Feltham, R. D. Principles of structure, bonding, and reactivity for metal nitrosyl complexes. *Coord Chem Rev.* **1974**, *13* (4), 339-406.

(13) Lewandowska, H.; Kalinowska, M.; Brzóska, K.; Wójciuk, K.; Wójciuk, G.; Kruszewski, M. Nitrosyl iron complexes—synthesis, structure and biology. *Dalton Trans.* **2011**, *40* (33), 8273-8289.

(14) Truzzi, D. R.; Augusto, O.; Iretskii, A. V.; Ford, P. C. Dynamics of Dinitrosyl Iron Complex (DNIC) Formation with Low Molecular Weight Thiols. *Inorganic Chemistry* **2019**, *58* (19), 13446-13456.

(15) Reginato, N.; McCrory, C. T. C.; Pervitsky, D.; Li, L. Synthesis, X-ray Crystal Structure, and Solution Behavior of Fe(NO)2(1-MeIm)2: Implications for Nitrosyl Non-Heme-Iron Complexes with g = 2.03. *J Am Chem Soc.* **1999**, *121* (43), 10217-10218.

(16) Tsai, F.-T.; Kuo, T.-S.; Liaw, W.-F. Dinitrosyl Iron Complexes (DNICs) Bearing O-Bound Nitrito Ligand: Reversible Transformation between the Six-Coordinate {Fe(NO)2}9 [(1-MeIm)2(η2-ONO)Fe(NO)2] (g = 2.013) and Four-Coordinate {Fe(NO)2}9 [(1-MeIm)(ONO)Fe(NO)2] (g = 2.03). *J Am Chem Soc.* **2009**, *131* (10), 3426-3427.

(17) Tonzetich, Z. J.; Wang, H.; Mitra, D.; Tinberg, C. E.; Do, L. H.; Jenney, F. E., Jr.; Adams, M. W. W.; Cramer, S. P.; Lippard, S. J. Identification of Protein-Bound Dinitrosyl Iron Complexes by Nuclear Resonance Vibrational Spectroscopy. *J Am Chem Soc.* **2010**, *132* (20), 6914-6916.

(18) Pereira, J. C. M.; Iretskii, A. V.; Han, R.-M.; Ford, P. C. Dinitrosyl Iron Complexes with Cysteine. Kinetics Studies of the Formation and Reactions of DNICs in Aqueous Solution. *J Am Chem Soc.* **2015**, *137* (1), 328-336.

(19) Vanin, A. F. Why Does not Oxygen as an Oxidizer of Nitric Oxide Eliminate it from the Human and Animals' Organism? (The Role of EPR-Active Dinitosyl Iron Complexes with Thiol-Containing Ligands in the Preservation of Nitric Oxide in These Organisms). *Applied Magnetic Resonance* **2025**, *56* (12), 1595-1608.

(20) Gordon, J. B.; McGale, J. P.; Prendergast, J. R.; Shirani-Sarmazeh, Z.; Siegler, M. A.; Jameson, G. N. L.; Goldberg, D. P. Structures, Spectroscopic Properties, and Dioxygen Reactivity of 5- and 6-Coordinate Nonheme Iron(II) Complexes: A Combined Enzyme/Model Study of Thiol Dioxygenases. *J Am Chem Soc.* **2018**, *140* (44), 14807-14822.

(21) Tchesnokov, E. P.; Wilbanks, S. M.; Jameson, G. N. L. A Strongly Bound High-Spin Iron(II) Coordinates Cysteine and Homocysteine in Cysteine Dioxygenase. *Biochemistry* **2012**, *51* (1), 257-264.

(22) Tchesnokov, E. P.; Fellner, M.; Siakkou, E.; Kleffmann, T.; Martin, L. W.; Aloi, S.; Lamont, I. L.; Wilbanks, S. M.; Jameson, G. N. L. The Cysteine Dioxygenase Homologue from Pseudomonas aeruginosa is a 3-Mercaptopropionate Dioxygenase. *J Biol Chem.* **2015**.
